# Kv1.3 as an upstream regulator of oxidative stress-mediated neuroinflammation following organic dust exposure in murine *in vitro*, *ex vivo*, and *in vivo* models

**DOI:** 10.3389/ftox.2026.1765108

**Published:** 2026-03-24

**Authors:** Nyzil Massey, Sanjana Mahadev Bhat, Denusha Shrestha, Emir Malovic, Locke A. Karriker, Shivani Choudhary, Alan P. Robertson, Hai Minh Nguyen, Heike Wulff, Anumantha G. Kanthasamy, Chandrashekhar Charavaryamath

**Affiliations:** 1 Biomedical Sciences, Iowa State University, Ames, IA, United States; 2 Mayo Clinic, Rochester, MN, United States; 3 Department of Veterinary & Biomedical Science, South Dakota State University, Brookings, SD, United States; 4 University of Illinois Chicago, Chicago, IL, United States; 5 Veterinary Diagnostic and Production Animal Medicine (VDPAM), Iowa State University, Ames, IA, United States; 6 Department of Pharmacology, School of Medicine, University of California, Davis, Davis, CA, United States; 7 Isakson Center for Neurological Disease Research, Department of Physiology and Pharmacology, College of Veterinary Medicine, University of Georgia, Athens, GA, United States; 8 Department of Veterinary Biomedical Sciences, Shreiber School of Veterinary Medicine (SSVM), Rowan University, Glassboro, NJ, United States

**Keywords:** Kv1.3, microglia, neuroinflammation, organic dust (OD), PAP-1, phosphorylatedp38 MAPK (p-p38 MAPK)

## Abstract

**Introduction:**

Inhalation of organic dust from concentrated animal production operations induces inflammation in the brain and respiratory tract. We investigated the role of the potassium channel Kv1.3 in models of organic dust (OD)-induced neuroinflammation. Kv1.3 channels play multifaceted roles in microglial immune modulation, cancer, and neurodegenerative diseases, and represent a potential therapeutic target.

**Methods:**

We used in vivo (C57BL/6 mice), in vitro (microglial cell line, and primary microglia), and ex vivo (brain slice culture) models of OD-induced neuroinflammation. Sterile OD extract (ODE) was prepared, and mice were exposed to either normal saline or ODE intranasally for 5 weeks (5 days/week) to simulate an occupational exposure scenario. Primary microglia were isolated from neonatal mice for total RNA sequencing (RNA-seq).

**Results:**

The ODE-induced expression of Kv1.3 was quantified using in vitro and ex vivo models with and without PAP-1 treatment. Exposure-induced changes in cytokines and markers of reactive species were measured. Using western blot, we quantified phosphorylated p38 MAPK (p-p38 MAPK) and NOX2. We measured microglial Kv1.3 currents using whole-cell patch-clamp. Exposure to ODE increased the expression of Kv1.3 and p-p38 MAPK in mouse microglia without affecting the Kv1.3 currents at the cell surface. Exposure increased the levels of inflammatory cytokines and NOX2. Kv1.3 inhibition with PAP-1 decreased inflammatory markers (TNF-α in BV2 microglia and IL-6 in Brain slice cultures and BV2 microglia), levels of Kv1.3, p-p38 MAPK, NOX2, and nitrites.

**Conclusion:**

Our study revealed that pharmacological inhibition of Kv1.3 potassium channels reduces ODE-induced neuroinflammation by decreasing inflammatory and oxidative stress markers.

## 1 Introduction

Approximately one-quarter of the global workforce is engaged in agriculture (Roser, 2023) and with 170,000 deaths per year, farm work is one of the most dangerous professions (International Labor Organization, 2024). The increasing global population and ever-increasing demand for more affordable protein sources have transformed the animal food production sector into efficient large-scale production units. These animal production facilities are known to produce and store many contaminants, including organic dust (OD) and various irritant gases on site (Nordgren and Charavaryamath, 2018; Poole et al., 2024; Sethi et al., 2017). Inhaled OD is currently under investigation for its adverse effects, specifically its role in triggering respiratory and neurological inflammation (Massey et al., 2021; Massey et al., 2022; Poole et al., 2009). This dust comprises a complex mixture of gases such as methane, ammonia, and hydrogen sulfide (H_2_S), particulate matter (PM) ranging from 0.01 to 1,000 µm in size, and numerous pathogen-associated molecular patterns (PAMPs) such as lipopolysaccharide (LPS) and peptidoglycan (Nordgren and Charavaryamath, 2018; Poole et al., 2024). The majority of the current research on the health impacts of OD is focused on its respiratory effects (Bhat et al., 2019; Bhat et al., 2022; Mahadev Bhat et al., 2021; Poole et al., 2024; Schwab et al., 2024; Shrestha et al., 2021; Shrestha et al., 2022). At the same time, our group has started investigating how inhaled OD exposure leads to neuroinflammation (Massey et al., 2019; Massey et al., 2021; Massey et al., 2022). Despite these efforts, the cellular and molecular underpinnings of neuroinflammation due to exposure to OD remain largely elusive.

Research has established a link between farming activities in the United States and a heightened occurrence of neurodegenerative diseases, particularly in the Midwest region (Wright Willis et al., 2010). Supporting this, research conducted in Iowa (Arora et al., 2021) suggests that individuals employed in agriculture are more likely to develop dementia, implicating occupational exposure in agriculture as a key factor in triggering neuroinflammation. Several studies have described how inhaled diesel exhaust particles (DEP), pesticides, and PM can negatively impact brain health (Levesque et al., 2011; Sánchez-Santed et al., 2016; Roux et al., 2017). However, how respiratory exposure to OD leads to neuroinflammation and subsequent cognitive impairments is not fully understood.

Our previous work has demonstrated that exposure of mouse brain microglial cells to OD extract (ODE) triggers a pro-inflammatory response via HMGB1-RAGE signaling (Massey et al., 2019). Either blocking nucleocytoplasmic translocation of HMGB1 via ethyl pyruvate or siRNA-mediated reduction in HMGB1 expression curtailed the pro-inflammatory response (Massey et al., 2019). Additionally, our use of mitoapocynin (MA), a mitochondria-targeted NOX2 inhibitor (Ghosh et al., 2016) decreased the ODE-induced reactive nitrogen species (RNS) production (Massey et al., 2019). Our published work also highlights the role of mitochondrial dysfunction in exposure of human THP1 cells (a model of macrophages (Genin et al., 2015)) to ODE (Mahadev Bhat et al., 2021). Recently, using both *in vitro* microglial cell lines and *ex vivo* brain slice cultures, we have shown that ODE exposure results in mitochondrial DNA (mtDNA) leakage into the cytosol, suggesting a mitochondrial membrane compromise (Massey et al., 2021). This extramitochondrial mtDNA activates the cGAS-STING pathway, an innate immune mechanism for detecting foreign DNA (Wu et al., 2013). We have demonstrated that MA treatment and the siRNA-mediated reduction of STING mRNA greatly diminish the ODE-induced inflammatory response (Massey et al., 2021).

Our *in vitro* and *ex vivo* models of OD exposure have uncovered mechanisms of exposure-induced cellular inflammation and ultra-structural damage to mouse microglia (Massey et al., 2019; Massey et al., 2021; Massey et al., 2022). To provide translational relevance, we used an established mouse model that was originally designed by Dr. Poole’s group and later used by our group (Charavaryamath et al., 2005; Poole et al., 2009). Our results from the mouse experiments concluded that prolonged intranasal ODE exposure elicited an inflammatory response in the mouse brain, and treatment with oral MA provided a partial neuroprotection (Massey et al., 2022).

Given that microglia are the chief innate immune cells in the brain, understanding their response to ODE exposure is critical. Hence, an RNA sequence (RNA-seq) analysis of microglia will likely provide the transcriptomic details of the exposure-induced neuroinflammation. The current study began with ODE exposure to identify gene expression changes in mouse brain microglia through RNA-seq analysis. This initial analysis revealed differential expression patterns, highlighting upregulated pathways associated with biological processes (BP) and molecular functions (MF). These findings provided the basis for our subsequent investigation into the specific roles of the Kv1.3 and MAPK14 pathways. Voltage-gated potassium (Kv) ion-channels have recently been identified as key players in neuroinflammatory conditions and other disorders (Chen et al., 2021; Maezawa et al., 2018; Nelson, 2006; Sarkar et al., 2020). The *kcna3* gene that encodes for Kv1.3 is predominantly expressed in immune cells. Microglial cells express significant amounts of Kv1.3 channels (Nguyen et al., 2017) and play a central role in neuroinflammation and neurodegeneration (Castro-Gomez and Heneka, 2024; Chen et al., 2021; Chen et al., 2018; Krause and Müller, 2010; Maezawa et al., 2018; Nguyen et al., 2017).

We have previously shown that microglia, upon exposure to ODE, generate reactive oxygen species (ROS) and nitrite (Massey et al., 2019). The interaction between superoxide and nitrite results in the formation of peroxynitrite, a compound with neurotoxic properties. As a potent oxidizing and nitrating substance, peroxynitrite is implicated in the advancement of neurodegenerative diseases, primarily through its ability to induce neuronal death. Peroxynitrite is known to kill neurons via the Kv1.3 mediated microglial respiratory burst (Fordyce et al., 2005; Torreilles et al., 1999).

The Kv1.3 channels are required for LPS-induced microglia activation and neuroinflammation (Di Lucente et al., 2018; Fordyce et al., 2005). Since OD is a complex mixture containing LPS (Nordgren and Charavaryamath, 2018) and other toxicants, it can trigger a multifaceted signaling pathway. The p38-α subtype of mitogen-activated protein kinases (MAPK14) detects cellular damage, and via NF-κB pathway produces cytokines and RNS (Ashraf et al., 2014; Canovas and Nebreda, 2021; Fordyce et al., 2005). The phosphorylated form of MAPK14 (pMAPK14) is vital in the neurotoxic signaling in microglia (Fordyce et al., 2005). pMAPK14 influences the expression of Kv1.3 channels during HIV-1 glycoprotein 120 induced microglial neurotoxicity and Kv1.3 channels are a potential therapeutic target in neuroinflammation models (Liu et al., 2012). Moreover, PAP-1 (5-(4-phenoxybutoxy)psoralen) selectively inhibits Kv1.3 channels in a state-dependent manner without showing toxicity. Kv1.3 blockade with PAP-1 has been shown to reduce neuroinflammation and neurodegeneration in a Parkinson’s disease model (Sarkar et al., 2020). Based on these premises, we hypothesize that OD exposure increases the expression of Kv1.3 channels and pMAPK14, leading to increased ROS, nitrite, and inflammatory cytokine production.

In this manuscript, we first used an adult mouse model of intranasal ODE exposure and performed RNA-seq analysis of brain microglia. Our results confirmed that, ODE exposure upregulates Kv1.3 expression. Next, we tested our hypothesis using an *in vitro* microglial cell line, primary microglial cultures of neonatal mice, and an *ex vivo* brain slice culture (BSC) model. To clarify the role of Kv1.3 in OD-induced neuroinflammation, we utilized PAP-1 as a pharmacological inhibitor. We concluded that PAP-1-mediated targeting of Kv1.3 channels reduces the ODE-induced neuroinflammation by decreasing inflammatory and oxidative stress markers.

## 2 Methods and materials

### 2.1 Preparation of organic dust extract

All experiments were conducted in accordance with an approved protocol from the Institutional Biosafety Committee (IBC protocol# 19-004) of Iowa State University. Settled swine barn dust (representing organic dust, OD) was collected from various swine production units into sealed bags with a desiccant and transported on ice to the laboratory. Sample collection was conducted in collaboration with a swine medicine specialist from facilities housing approximately 1,000 market-weight pigs (75–295 lbs), maintained on dry grain diets with *ad libitum* access to food and water. Waste was managed using slatted concrete flooring over deep manure pits, and mechanical ventilation systems operated under negative pressure. Most collections occurred during winter months when ventilation rates were lowest. Dust samples were pooled from multiple sites and used to prepare the organic dust extract (ODE). Inflammatory potential was previously validated by assessing cytokine secretion from macrophages and microglia. Endotoxin concentrations in these pooled samples ranged from 0.81 to 1.43 EU/mL, as previously reported, and are assumed to be within this range in the current study (Bhat et al., 2022). Organic dust extract (ODE) was prepared as per a published protocol (Romberger et al., 2002). Briefly, dust samples were weighed, and for every gram of dust, 10 mL HBSS, no calcium, no magnesium (Thermo Fisher Scientific, cat. # 14170112) was added, stirred, and allowed to stand at room temperature for 60 min. The mixture was centrifuged (1,365 g, 4 °C) for 20 min, the supernatant collected, and the pellet was discarded. The supernatant was centrifuged again with the same conditions, the pellet discarded, and the recovered supernatant was filtered using a 0.22 µm filter and stored at −80 °C until used. This stock was considered 100% and diluted in cell culture medium to prepare a 1% v/v solution to use in our *in vitro* experiments (Table 1). We routinely quantify the LPS content of our ODE samples using Pyrochrome® Kinetic Chromogenic Endotoxin Assay (Cape Cod, cat. # CG-1500-5) as per the instructions. We have previously published the LPS content of several of our ODE samples (Bhat et al., 2019). However, we did not measure the LPS content of the ODE samples used in the current experiments, including those in this manuscript.

**TABLE 1:** Treatments.

Treatment group	*in vivo*	*ex vivo*	*in vitro*
Control	Normal saline (Intranasal)	BSCs media	BV2 and primary microglia media
ODE	12.5% (5-day x 5 weeks) (Intranasal)	1% v/v (5 days)	1% v/v (48 h)
ODE + PAP-1	Not done	1 µM PAP-1 (co-treatment)	1 µM PAP-1 (co-treatment)
Lipopolysaccharide (positive control)	Not done	Not done	100 ng/mL

### 2.2 Chemicals and reagents for *in-vitro* and *ex-vivo* culture

Dulbecco’s Minimum Essential Medium (DMEM) (Thermo Fisher, cat. # 11965092), Penicillin-Streptomycin (10,000 U/mL) (Thermo Fisher, cat. # 15140122), L-Glutamine (200 mM) (Thermo Fisher, cat. # 25030081), and Trypsin-EDTA (0.25%), phenol red (Thermo Fisher, cat. # 25200072), and Fetal Bovine Serum (FBS) (Atlanta Biologicals, Flowery Branch, GA, cat. #S11150H, Lot # A17002) were utilized for *in vivo* and *ex vivo* culture. PAP-1 [5-(4-phenoxybutoxy) psoralen] was synthesized in Dr. Wulff’s laboratory as described (Schmitz et al., 2005) and reconstituted into a 10 mM/L stock solution in DMSO and stored at - 20 °C. PAP-1 was used as one of the co-treatments with ODE (1% v/v) (Table 1).

### 2.3 Animal care, treatment, and euthanasia

Mouse exposure experiments were performed per the approved protocol (IACUC-19-250) by the Institutional Animal Care and Use Committee (IACUC) at Iowa State University, Ames, IA, United States. Eight-week-old male C57BL/6 mice (3 mice/group), obtained from Charles River, were housed under the following standard conditions: constant temperature (22 °C ± 1 °C), humidity (relative, 30%), and a 12-h light/dark cycle. The animals were assigned randomly by a coin flip method to the treatment groups using the mean weight of the group as the criterion. The mean weight of each group was not significantly different from the other groups. Investigators involved in data collection and analysis were blinded to the treatment groups. After acclimatization for 7 days (week 0), mice were either intranasally administered normal saline (0.9% w/v) or 12.5% ODE (25 µL into each nostril, total of 50 µL/mouse/day) 5 days/week (Monday-Friday) for a total of 5 weeks (Table 1). To minimize stress, pain, and ensure accurate delivery of the extract, mice were briefly anesthetized with isoflurane for approximately 20 s prior to ODE instillation. Mice were euthanized following 5 weeks of intranasal exposure, using a pressurized CO_2_ chamber (AVMA-approved method), and freshly dissected brains were immediately harvested in ice-cold PBS. All the treatments were performed as per our previously published work (Massey et al., 2022).

### 2.4 Preparation of microglial single-cell suspension and myelin removal from the adult mouse brain

Meninges were removed from adult mice brains and the brains were cut into small pieces (<1 mm) with a scalpel and then placed in ice-cold Dulbecco’s modified Eagle’s medium/F-12 nutrient mixture DMEM/F-12 (Thermo Fisher, cat. # 3625) supplemented with 10% heat-inactivated fetal bovine serum (FBS), 50 U/mL penicillin, 50 μg/mL streptomycin, 2 mM L-glutamine, 100 μM non-essential amino acids, and 2 mM Sodium Pyruvate (Thermo Fisher, cat. # 11360070) also referred to as sample preparation medium. The tissue pieces were incubated after adding 0.25% trypsin-EDTA in a 37 °C water bath for 30 min with gentle agitation. A single-cell suspension of the digested brain tissue was prepared by gentle trituration and passing through 70 μm reversible Strainers (STEMCELL Technologies, cat. #27260) to remove tissue debris and aggregates. Single-cell suspension was centrifuged at 300 × g for 10 min at room temperature or at 2 °C–8 °C, with the brake on low. The supernatant was carefully removed and discarded. 30% Percoll® (GE Healthcare, cat.#. 17-0891-01) solution was added to the pellet and centrifuged at 700 × g for 10 min at room temperature or 2 °C–8 °C. The upper myelin layer was carefully removed and discarded. The pellet was washed with sample preparation medium once and resuspended to the final concentration of 2.5 × 10^7^ cells/mL.

### 2.5 Column-free microglial isolation from single-cell suspension (adult mice and neonatal mice)

Microglia were isolated from freshly dissected neonatal and adult mouse brains, depending on the experimental paradigmvia EasySep mouse CD11b positive selection kit II (STEMCELL Technologies, cat. # 18970). The single-cell suspension was resuspended at a density of up to 2.5 × 10^7^ cells/mL in the separation medium (PBS containing 2% FBS and 1 mM EDTA, with no calcium or magnesium) and transferred to a 5 mL polystyrene round-bottom tube (Corning, cat. # 352058). The microglia were then isolated as described in a previous study (Gordon et al., 2011). Anti-CD16/32; FcR blocker (BioLegends, cat. # 101301) was used to prevent non-specific labeling of other cell types and thus enhance the purity of the single-cell suspension. To confirm the purity of isolated primary microglia, we performed Immunocytochemistry validation using Iba1, a specific marker for microglia. Magnetically isolated cells were seeded on poly-D-lysine-coated coverslips, stained with anti-Iba1 antibody, and counterstained with DAPI. Quantification using ImageJ revealed that over 96% of DAPI^+^ cells were also Iba1^+^, confirming high purity of the microglial preparation (see Supplementary Figure 4). Furthermore, cell viability was assessed using the Vi-CELL XR automated cell counter (Beckman Coulter), which employs the trypan blue exclusion method. Our preparations consistently showed >90% viability.

### 2.6 Mammalian cell culture and treatments (BV2 microglia and primary microglia)

BV2 microglial cell line derived from wild-type C57BL/6 mice (Halle et al., 2008) was a kind gift from Dr. DT Golenbock (University of Massachusetts Medical School, Worcester, MA) to Dr. AG Kanthasamy. Microglial cells **(**BV2 microglia and primary microglia) were grown in T-75 flasks (1 × 10^6^ cells/flask), 12-well tissue culture plates coated with poly-D Lysine, and 24-well tissue culture plates (50 × 10^3^ cells/well). Cells were maintained in DMEM supplemented with 10% heat-inactivated fetal bovine serum (FBS), 50 U/mL penicillin, 50 μg/mL streptomycin, and 2 mML-glutamine. Cells were incubated overnight before treatment. All the treatment group details are outlined in Table 1. Control group samples were collected at 0 h because the control group samples from 6, 24, and 48 h time points showed no differences in our pilot studies (Massey et al., 2019). All treatments were done for 48 h as per previous publications (Massey et al., 2019; Massey et al., 2021). LPS was used as a positive control for microglial stimulation (Table 1).

### 2.7 RNA sequencing data quantification and analysis of isolated microglia from adult mice’s brains

Total RNA was extracted from isolated microglia using TRIzol reagent, and quality was assessed using an Agilent 2100 Bioanalyzer (Agilent Technologies, Santa Clara, CA, United States). After library preparation, RNA sequencing was performed at Iowa State University’s DNA Facility. Raw reads in fastq.gz format from each sample were processed using Galaxy (https://usegalaxy.org/), an open-source platform for bioinformatics workflows (Galaxy Community, 2024). The initial step involved obtaining clean, high-quality reads by removing adapter sequences and filtering low-quality reads. This step was performed using Trim Galore! (Galaxy version 0.6.10). Quality metrics such as Q20, Q30, and GC content were assessed using FastQC (Galaxy version 0.73+galaxy0) and MultiQC (Galaxy version 1.17+galaxy0) for clean reads. The high-quality reads were aligned to the mouse reference genome (e.g., GRCm39) using HISAT2 (Galaxy version 2.2.1+galaxy0). The genome reference files (FASTA and GTF annotation) were downloaded from GENCODE and uploaded to Galaxy. The GENCODE is an encyclopedia of genes and gene variants from the National Human Genome Research Institute (NHGRI).

Next, single-end alignment was performed with default settings, generating BAM files for each sample. BAM files contained chromosomal coordinates for the mapped reads. Mapped reads were assigned to genes using featureCounts (Galaxy version 2.0.3+galaxy1), a tool from the Subread package. It utilized built-in annotations to calculate read counts based on genomic start and end positions of each exon. Individual count files were generated for each sample, and a count matrix was created using Concatenate datasets (Galaxy version 1.0.0) to consolidate counts into a single table (genes in rows, samples in columns). The count matrix, gene annotation file (containing gene symbols and descriptions), and factor data file (e.g., Control vs. ODE) were input into the limma-voom pipeline implemented via the limma-voom (Galaxy version 3.50.0+galaxy0) tool. The voom function applies precision weights to the raw counts data, and edgeR normalization was used to calculate counts-per-million (CPM) values (Robinson et al., 2010). Genes with an adjusted *p* ≤ 0.05 (corrected for multiple testing using the false discovery rate, FDR) were identified as differentially expressed genes (DEGs).

RNA-seq analysis was conducted as an exploratory, hypothesis-generating approach to identify transcriptional changes associated with ODE exposure. Differential expression was assessed using gene-level summary statistics, including log fold change, average expression, and associated statistical values. Key differentially expressed genes identified through RNA-seq were subsequently validated by independent qPCR analysis to support the robustness of the transcriptomic findings.

### 2.8 DAVID enrichment and gene ontology (GO) analyses of DEGs

Lists of differentially expressed genes (DEGs) and their corresponding ENTREZ IDs (*p* ≤ 0.05) for each treatment group were uploaded to the DAVID (Database for Annotation, Visualization, and Integrated Discovery) tool. The species background was set to “*Mus musculus*”. Gene Ontology (GO) enrichment analyses were conducted to identify significantly enriched terms within the categories of biological processes (BP), molecular functions (MF), and cellular components (CC). The DAVID pathway viewer was utilized to explore and visualize these enriched pathways, providing functional annotation and insights into the biological significance of the DEGs.

### 2.9 Brain slice cultures (BSCs)

The research outlined in this document adhered to the protocols sanctioned by the Institutional Animal Care and Use Committee of Iowa State University (IACUC protocol #18-290). The methodology for preparing mouse organotypic brain slices was in line with established procedures (Kondru et al., 2017). Following an approved animal breeding protocol (IACUC protocol #18-227), breeding pairs of C57BL/6 mice were obtained from The Jackson Laboratories (Bar Harbor, ME), and mating was initiated when the mice were approximately 4 weeks old. Offspring were nurtured by their parents until they reached the age of 9–12 days. At this stage, both male and female pups were humanely euthanized via cervical dislocation, a method endorsed by the AVMA for animals within this age range. The brain slices were then carefully prepared from the freshly harvested brain matter using a Compresstome™ VF-300 microtome (Precisionary Instruments). This process involved positioning the entire brain along the mid-sagittal plane inside the Compresstome’s tube, previously filled with 2% agarose gel. The gel was rapidly set by cooling with a chilling block. Subsequently, the tube was placed into a slicing chamber containing a cold solution of Gey’s balanced salt solution enhanced with kynurenic acid (GBSSK) to prevent excitotoxicity. The GBSS was concocted by sequentially mixing components from concentrated stocks to achieve the desired final concentrations per liter: NaCl (8 g), KCl (0.37 g), Na_2_HPO_4_ (0.12 g), CaCl_2_∙2H_2_O (0.22 g), KH_2_PO_4_ (0.09 g), MgSO_4_∙7H_2_O (0.07 g), MgCl_2_∙6H_2_O (0.210 g), and NaHCO_3_ (0.227 g). The Compresstome’s compression lip, situated in the cutting area, ensured the brain remained stable during the slicing process, which yielded 350-μm thick sections at a medium vibration setting. These BSCs were collected from the tube’s exit and relocated to a new plate containing fresh GBSSK. After their preparation, the brain slice cultures (BSCs) underwent a double wash in 6 mL of chilled GBSSK. Slices were then placed into 6-well plate inserts (Falcon, catalog #353090), each containing 3 to 4 slices, and incubated at 37 °C in a 5% CO_2_ humidified environment for a fortnight. The process of slicing brain tissue can damage neuronal and glial cells, leading to immediate gliosis post-preparation. To mitigate this problem, a pre-treatment incubation period for the BSCs is advised. We performed a 2-week incubation that has been shown to alleviate sectioning-induced trauma (Croft and Noble, 2018; Kondru et al., 2017). During this period, the culture medium was refreshed bi-daily. Following the incubation, a 5-day treatment was administered to the BSCs (Table 1).

### 2.10 Western blot analysis

Isolated microglia from fresh brain tissue samples were collected from animals after dissection and placed in RIPA buffer. Isolated microglia were further triturated into a suspension and sonicated using a sonication bath for 60 s with 50% power, 5 s of pulses, and 3 s of resting periods to get a whole cell lysate. Total protein was estimated using Bradford assay. Equal amounts of proteins (20 μg/well) were resolved on 10% SDS-PAGE gels (Bio-Rad). Next, proteins were transferred to a nitrocellulose membrane, and the nonspecific binding sites were blocked for an hour with a blocking buffer specially formulated for fluorescent Western blotting (Rockland Immunochemicals, Pottstown, PA). Membranes were incubated overnight at 4 °C with the respective primary antibodies (listed in Table 2). Next, membranes were incubated with the respective secondary donkey anti-rabbit IgG highly cross-adsorbed (A10043) or anti-mouse 680 Alexa Fluor antibodies (A21058, Thermo Fisher Scientific). Membranes were washed three times with PBS containing 0.05% Tween-20 and visualized on the Odyssey infrared imaging system. Band densities were normalized using β-actin (1:5000, Abcam; ab6276 or ab8227) as a loading control, and densitometry was performed (ImageJ, NIH).

**TABLE 2:** Antibodies used in Western blotting.

Primary target	Catalog #	Vendor
KCNA3/kv1.3	APC-101	Almone labs
pMAPK14/p-p38 MAPK	9211S	Cell signaling

### 2.11 qPCR analysis

Total RNA from mice brains, BSCs, and cell culture samples was isolated using TRIzol™ (Invitrogen, cat. # 15596-026) as per the manufacturer’s guidelines. RNA concentration was measured using NanoDrop, and the A260/A280 ratio was used to determine the RNA quality. Samples with an A260/A280 ratio (1.8–2.1) were considered acceptable and used for further analysis. 1 μg of RNA was reverse transcribed into cDNA using the superscript IV VILO Kit (Thermo Fisher Scientific, cat. # 11766050) following the manufacturer’s protocol. For qPCR, 5 µL of PowerUpTM SYBR Green Master mix (Thermo Fisher Scientific, cat. # 25742), 0.5 µL each of forward and reverse primers (10 µM), 3 µL of water and 1 µL of cDNA (1–10 ng) were used. The genes and their respective primer sequences used for qPCR analysis are listed in Supplementary Table 3 for DEGs validation (*in vivo*) and Gene expression (*in vitro* and *ex vivo*). All primers were synthesized at Iowa State University’s DNA Facility and β-actin was used as a housekeeping gene. No template controls and dissociation curves were run for all experiments to exclude cross-contamination. CT values of the gene products of interest were normalized to housekeeping gene product CT values. Comparisons were made between experimental groups using the ΔCT method. Briefly, the ΔCT value was calculated for each sample (CT gene of interest minus CT β-actin). Then the calibrator value was averaged (ΔCT) for the control samples. The calibrator was subtracted from the ΔCT for each control and from the experimental sample to derive the ΔΔCT. The fold change was calculated as 2^−ΔΔCt^ (Livak and Schmittgen, 2001). The average fold change was calculated for each experimental group and for the full names of each gene, please refer to the supplementary data (Supplementary Table 4).

### 2.12 Cytokine analysis by ELISA

Culture media was collected following treatment of BSCs (5 days) and analyzed for TNF-α (Thermo Fisher Scientific, cat. # 88-7324-88) and IL-6 levels (Thermo Fisher Scientific, cat. # 88-7064-77) using ELISA kits by following the recommended manufacturer’s protocols.

### 2.13 Luminex assays for cytokine detection in BV2 microglia cell line and primary microglia of neonatal mice

TNF-α, IL-6, IL-1β, IL-10, IL-17A, and IFNγ levels were assessed in supernatant culture media via Luminex assays. 40μL of neat media was added to 40 μL of primary antibody conjugated to magnetic microspheres and incubated overnight at 4 °C in a clear-bottom, black 96-well plate. After incubation, each well was washed (×3) using a magnetic washer and then incubated for 1 hour with secondary antibodies. Finally, samples were incubated for 30 min with streptavidin/phycoerythrin. A Bio-Plex reader was used to read the 96-well plates at 485 nm for excitation and 520 nm for emission. A standard curve of all the cytokines was prepared using standard cytokines.

### 2.14 Detection of nitrite levels using the griess reagent

We measured nitrite production (RNS marker) in BV2 microglia and BSCs using Griess reagent (Sigma-Aldrich). Briefly, the supernatant was collected following treatments (Table 1). Griess reagent was added to 50 µL of supernatant samples in a 98-well plate (1:1) and incubated at room temperature for 10 min until color development. A standard nitrite curve was prepared from sodium nitrite. The color change was analyzed by measuring absorbance at 540 nm using a microplate reader (Spectramax M2, Molecular Devices). The absorbance values were expressed in µM, and data was analyzed using GraphPad Prism software.

### 2.15 Immunohistochemistry (IHC) analysis in BSCs

Brain slice cultures (BSCs) were prepared from mouse pups (n = 3 per group), with slices derived from different animals rather than repeated sampling from a single pup, and treated with ODE or control conditions for 5 days as previously described (Massey et al., 2021). All treatment groups are listed in Table 1. Following treatment, BSCs on inserts were carefully excised using a scalpel and transferred to new 12-well inserts with the tissue facing upward. Slices were washed twice with PBS, fixed in 4% paraformaldehyde at room temperature for 30 min, and incubated with ice-cold 20% methanol in PBS for an additional 5 min. Permeabilization was performed using 1% Triton X-100 in PBS at 4 °C for 12–18 h. To minimize non-specific staining, BSCs were blocked with 20% BSA containing 0.1% Triton X-100 in PBS for 2–3 h and incubated overnight at 4 °C with primary antibodies (Table 3). After three washes with 5% BSA in PBS, BSCs were incubated with secondary antibodies for 12 h at 4 °C. Following secondary antibody incubation, slices were mounted using VECTASHIELD antifade mounting medium containing DAPI (Vector Laboratories, Burlingame, CA), coverslipped, and sealed with nail polish. Imaging was performed using a Nikon Eclipse TE2000-U microscope, and images were acquired with a Photometrics CoolSNAP CF camera using identical acquisition settings (exposure time, gain, and illumination intensity) across all experimental groups. Fluorescence images were processed using HCImage software (Tucson, AZ). Quantitative image analysis was performed using standardized thresholding and background subtraction parameters applied uniformly across conditions, with fluorescence intensity normalized to cell number or area as appropriate. Representative images were selected from fields consistent with the quantified datasets.

**TABLE 3:** Antibodies used for immunostaining.

Primary target	Catalog #	Vendor
Kv1.3	APC-101	Almone labs
pMAPK14	9211S	Cell signaling
NOX2	Ab129068	Abcam
IBA1	ab5076	Abcam

### 2.16 Immunocytochemistry (ICC) analysis in BV2 microglia and primary microglia of neonatal mice

BV2 microglia and primary microglia were seeded onto three 35-mm glass coverslips per condition placed in 24-well culture plates and maintained in DMEM (Thermo Fisher Scientific, Waltham, MA) supplemented with 10% heat-inactivated FBS, 50 U/mL penicillin, 50 μg/mL streptomycin, and 2 mM L-glutamine. Cells were allowed to adhere overnight and then treated as outlined in Table 1. Following treatment, cells were washed with ice-cold DPBS and blocked using 10% normal donkey serum (EMD Millipore, Burlington, MA) containing 0.2% Triton X-100 in PBS for 1 h at room temperature. Primary antibodies (Table 3) were prepared in antibody diluent solution (2.5% normal donkey serum, 0.25% sodium azide, 0.2% Triton X-100 in PBS; Abcam, Cambridge, MA), and cells were incubated with primary antibodies overnight at 4 °C. After primary antibody incubation, cells were washed five times with PBS and incubated with the appropriate secondary antibodies for 1 h at room temperature. Coverslips were mounted onto glass slides using VECTASHIELD antifade mounting medium containing DAPI (Vector Laboratories, Burlingame, CA) and allowed to dry overnight. Imaging was performed using a Nikon Eclipse C1 confocal microscope, with identical acquisition settings (laser power, detector gain, and exposure time) applied across all experimental groups. Images were processed using standardized thresholding and background subtraction parameters, applied uniformly across conditions, and fluorescence intensity was normalized to cell number as appropriate. Representative images were selected from fields consistent with those used for quantitative analysis.

### 2.17 IHC and ICC quantification

Five fields per coverslip were chosen randomly, and total stained cells were counted (ImageJ). Total staining intensity per field (cy3 or FITC) was measured using HCImage software (Hamamatsu Corporation, Sewickley, PA). The mean cell intensity was measured by dividing total intensity by the number of cells per field (Massey et al., 2019). The average mean intensity for each coverslip was calculated and plotted.

### 2.18 Whole-cell patch-clamping

Primary neonatal mouse microglia were plated at a density of 100,000–150,000 cells/well in 24-well plates for 8–12 h before stimulation with either 100 ng/mL LPS or 1% ODE. After 48-h, cells were detached by trypsinization, washed, attached to poly-D-lysine-coated glass coverslips, and then studied within 20–90 min after plating in the whole-cell mode of the patch-clamp technique with an EPC-10 HEKA amplifier (HEKA Elektronik). All currents were recorded in normal Ringer’s solution containing 160 mM NaCl, 4.5 mM KCl, 2 mM CaCl_2_, 1 mM MgCl_2_, and 10 mM HEPES (adjusted to pH 7.4 and 290–310 mOsm). Patch pipettes were pulled from soda-lime glass (micro-hematocrit tubes, Kimble Chase) to resistances of 2–3 MΩ when submerged in the bath solution and filled with a KF-based Ca^2+^-free internal pipette solution containing 145 mM KF, 2 mM MgCl_2_, 10 mM HEPES, and 10 mM EGTA (pH 7.2, 290–310 mOsm). Recording conditions were set up to isolate Kv1.3 currents. The use of KF avoids “contaminating” Kv currents with calcium-activated K^+^ currents or chloride currents. Currents were elicited with a “use-dependence protocol,” involving a train of ten 200-ms voltage steps from −80 to 40 mV applied at a frequency of 1 Hz, which identifies Kv1.3 by its characteristic use dependence (e.g., current amplitude declines rapidly when pulsed faster than the channels can recover from inactivation). In 25% of the cells, we then further pharmacologically confirmed that the current was predominantly carried by Kv1.3 by testing its sensitivity to PAP-1. Cell capacitance, which directly measures the cell-surface area, and access resistance were continuously monitored during recordings. Kv1.3 current density was calculated as the use-dependent current amplitude at 40 mV divided by the cell capacitance measured for individual cells (Nguyen et al., 2017).

### 2.19 Statistical analysis

**FIGURE 1:**
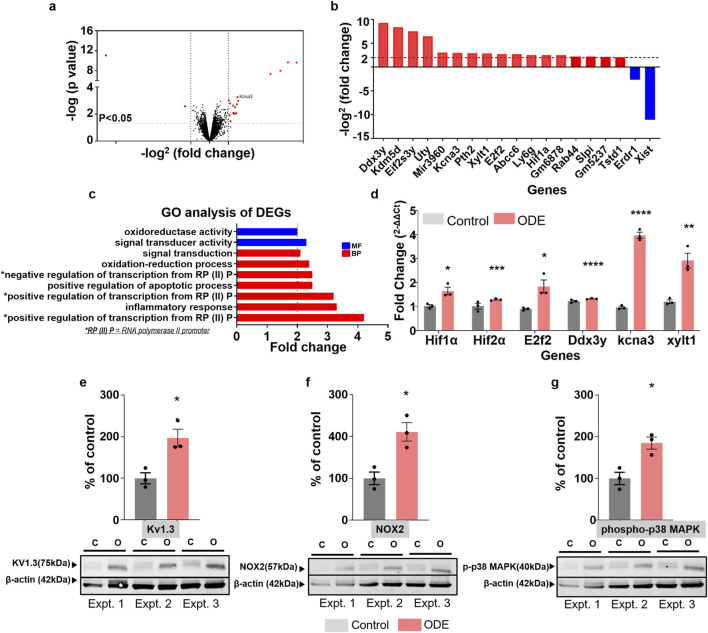
Transcriptomic profiling, qPCR validation, and protein expression analyses reveal microglial responses to ODE exposure in mouse brains. After 5 weeks of exposure to organic dust extract (ODE), brains were collected from male mice (n = 3/group), and the microglial fraction was isolated. Total RNA was extracted from isolated microglia for RNA-seq analysis, and differentially expressed genes (DEGs) were identified based on statistical significance (p ≤ 0.05) and a log_2_ fold-change >2. These DEGs are visualized using a volcano plot and bar graph **(a,b)**. Pathway enrichment analysis using DAVID focused on gene ontology (GO) terms, highlighting significantly enriched pathways related to biological processes (BP) and molecular functions (MF) **(c)**. Validation of selected DEGs was performed via qPCR analysis. From 40 genes initially selected (Supplementary Table 4), six genes demonstrated significant validation (log_2_ fold-change >2) compared to controls: Hypoxia-inducible factor 1α (Hif1α), Hypoxia-inducible factor 2α (Hif2α), E2F transcription factor 2 (E2f2), DEAD (Asp-Glu-Ala-Asp) box polypeptide 3, Y-linked (Ddx3y), Potassium voltage-gated channel (Kcna3), and Xylosyltransferase 1 (Xylt1) **(d)**. Additionally, Western blot analysis was conducted on whole-cell lysates of isolated microglia from freshly dissected mouse brains to assess protein expression levels of Kv1.3 (75 kDa), NOX2 (57 kDa), phosphorylated-p38 MAPK14 (p-p38 MAPK14, 40 kDa), and β-actin (housekeeping protein, 42 kDa). Densitometric quantification revealed significantly increased expression levels of Kv1.3, NOX2, and p-p38 MAPK14 proteins following ODE exposure compared to controls **(e–g)**. Please refer to Supplementary Figure 5 for ImageJ based densitometry analysis. Statistical significance is indicated by an asterisk (* for the ODE exposure effect, control vs. ODE). *p < 0.05, **p < 0.01, ***p < 0.001, ****p < 0.0001 by one-way ANOVA with Tukey’s multiple comparisons.

**FIGURE 2:**
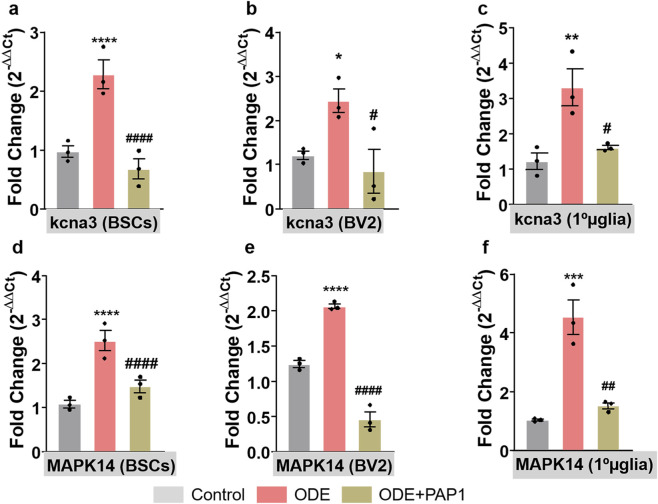
Regulation of kcna3 and MAPK14 gene expression by PAP-1 in ODE-treated BSCs, BV2 microglia, and primary microglia. qPCR analysis was performed on total RNA (free from genomic DNA) extracted from BSCs, BV2 microglia, and primary microglia (referred to as 1°µglia) to evaluate the expression of Kcna3 **(a)** and MAPK14 **(b)** genes. Specifically designed primers used for this analysis are listed in Supplementary Table 3. The ODE-exposed group exhibited significantly elevated mRNA expression of Kcna3 **(a–c)** and MAPK14 **(d–f)** compared to the control group. In contrast, the PAP-1 co-treatment group showed significantly reduced mRNA expression levels of both Kcna3 **(a–c)** and MAPK14 (d-f) in BSCs, BV2 microglia, and primary microglia. The sample size included n = 3 mouse pups for BSC and primary microglia; and n = 3 independent cultures for BV2 microglia (biological replicates). Statistical significance is indicated either by * or by # ( * for ODE exposure effect, control vs. ODE, and # for PAP-1 treatment effect, ODE vs. ODE + PAP1). *^/#^p < 0.05, **^/##^p < 0.01, ***^/###^p < 0.001, ****^/####^p < 0.0001 by one-way ANOVA with Tukey’s multiple comparisons.

**FIGURE 3:**
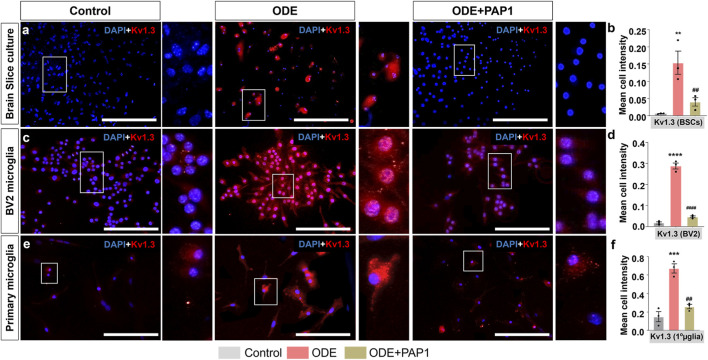
IHC and ICC analysis of Kv1.3 expression in ODE-treated BSCs, BV2 microglia, and primary microglia with PAP-1 treatment effects. Following the treatments (Table 1), BSCs **(a)**, BV2 microglia **(b)**, and primary microglia (referred to as 1°µglia) **(c)** were immunostained for Kv1.3 (Cy3, red). The antibodies used for immunostaining are detailed in Table 3. Nuclei were counterstained with DAPI (blue) to visualize and count the number of cells in each field. To specifically identify microglia expressing Kv1.3 in BSCs, co-staining with Iba1 (a microglia marker) was performed. Images of Iba1, FITC (green) panel (not included in the main manuscript) are provided in Supplementary Figure 1. Quantification of IHC/ICC staining for Kv1.3 expression was carried out as described in the Methods section. Compared to the control group, ODE-exposed groups showed a significant increase in Kv1.3 staining intensity in BSCs (b), BV2 microglia **(d)**, and primary microglia (referred to as 1°µglia) **(f)**. Notably, PAP-1 treatment significantly reduced Kv1.3 expression in BSCs **(a,b)**, BV2 microglia **(c,d)**, and primary microglia **(e–f)**, as shown in the representative images and quantitative analysis. The sample size included n = 3 mouse pups for BSC and primary microglia, and n = 3 independent cultures for BV2 microglia (biological replicates). Statistical significance is indicated by an * for the ODE exposure effect (control vs. ODE) and # for the PAP-1 treatment effect (ODE vs. ODE + PAP1). The scale bar represents 50 μm *^/#^p < 0.05, **^/##^p < 0.01, ***^/###^p < 0.001, ****^/####^p < 0.0001 by one-way ANOVA with Tukey’s multiple comparisons.

**FIGURE 4:**
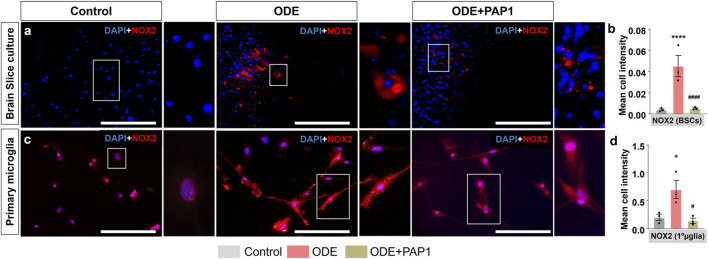
Immunocytochemistry analysis of NOX2 expression in ODE-treated BSCs, and primary microglia with PAP-1 treatment effects. Following the treatments (Table 1), BSCs **(a)**, and primary microglia (referred to as 1°µglia) **(c)** were immunostained for NOX2 (Cy3, red). The antibodies used for immunostaining are detailed in Table 3. Nuclei were counterstained with DAPI (blue) to visualize and count the number of cells in each field. To specifically identify microglia expressing NOX2 in BSCs, co-staining with Iba1 (a microglia marker) was performed. Images of Iba1, FITC (green) panel (not included in the main manuscript) are provided in Supplementary Figure 2. Quantification of immunohistochemistry (IHC) and immunocytochemistry (ICC) staining for NOX2 expression was carried out as described in the Methods section. Compared to the control group, ODE-exposed groups showed a significant increase in NOX2 staining intensity in BSCs **(b)**, and primary microglia (referred to as 1°µglia) **(d)**. Notably, PAP-1 treatment significantly reduced NOX2 expression in BSCs **(a,b)**, and primary microglia **(c,d)**, as shown in the images and quantitative analysis. The sample size included n = 3 mouse pups for BSC and primary microglia. Statistical significance is indicated by (* for ODE exposure effect, control vs. ODE, and # for PAP-1 treatment effect, ODE vs. ODE + PAP1). The scale bar represents 50 μm *^/#^p < 0.05, **^/##^p < 0.01, ***^/###^p < 0.001, ****^/####^p < 0.0001 by one-way ANOVA with Tukey’s multiple comparisons.

**FIGURE 5:**
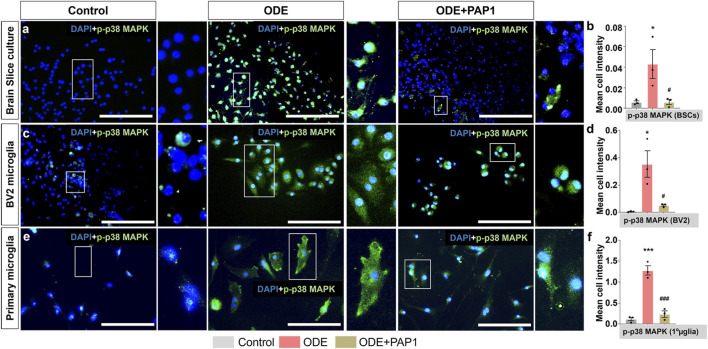
Immunocytochemistry analysis of p38 MAPK phosphorylation in ODE-treated BSCs, BV2 microglia, and primary microglia with PAP-1 treatment effects. Following the treatments (Table 1) BSCs **(a)**, BV2 microglia **(b)**, and primary microglia (referred to as 1°µglia) **(c)** were immunostained for p-p38 MAPK (FITC, green). The antibodies used for immunostaining are detailed in Table 3. Nuclei were counterstained with DAPI (blue) to visualize and count the number of cells in each field. To specifically identify microglia expressing p38 MAPK phosphorylation in BSCs, co-staining with Iba1 (a microglia marker) was performed. Images of Iba1, FITC (green) panel (not included in the main manuscript) are provided in Supplementary Figure 3. Quantification of immunohistochemistry (IHC) and immunocytochemistry (ICC) staining for p-p38 MAPK expression was carried out as described in the Methods section. Compared to the control group, ODE-exposed groups showed a significant increase in p38 MAPK phosphorylation in BSCs **(b)**, BV2 microglia **(d)**, and primary microglia (referred to as 1°µglia) **(f)**. Notably, PAP-1 treatment significantly reduced p38 MAPK phosphorylation in BSCs **(a,b)**, BV2 microglia **(c,d)**, and primary microglia **(e,f)**, as shown in the representative images and quantitative analysis. The sample size included n = 3 mouse pups for BSC and primary microglia, and n = 3 independent cultures for BV2 microglia (biological replicates). Statistical significance is indicated by (* for ODE exposure effect, control vs. ODE, and # for PAP-1 treatment effect, ODE vs. ODE + PAP1). The scale bar represents 50 μm *^/#^p < 0.05, **^/##^p < 0.01, ***^/###^p < 0.001, ****^/####^p < 0.0001 by one-way ANOVA with Tukey’s multiple comparisons.

**FIGURE 6:**
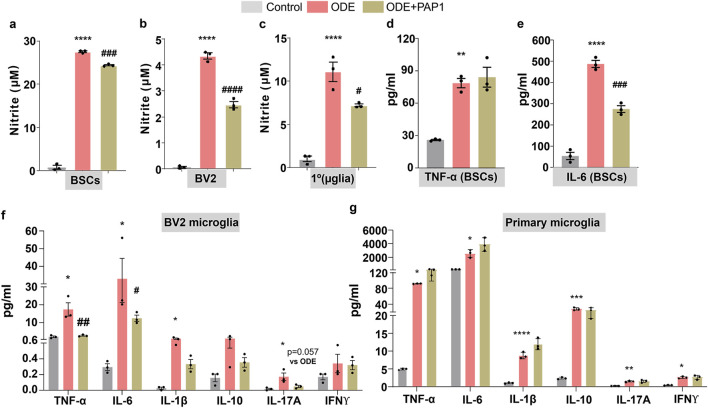
ODE-induced nitrite and cytokine responses in brain slice cultures, BV2, and primary microglia, and their modulation by PAP-1. The effects of ODE exposure and PAP-1 treatment on nitrite and cytokine secretion were evaluated in BSCs, BV2 microglia, and primary microglia. Nitrite concentrations in culture supernatants were quantified using the Griess assay **(a–c)**. ODE exposure significantly increased nitrite levels in BSCs **(a)**, BV2 microglia **(b)**, and primary microglia **(c)**, while co-treatment with PAP-1 markedly reduced these levels. Pro-inflammatory cytokines in BSC culture supernatants were analyzed using commercial ELISA kits **(d,e)**. ODE exposure elevated TNF-α **(d)** and IL-6 **(e)** levels, with PAP-1 significantly attenuating IL-6 but not TNF-α. Cytokine levels in BV2 and primary microglia were assessed using a customized MILLIPLEX® Mouse Cytokine/Chemokine Kit (f-g). In BV2 microglia **(f)**, ODE significantly increased TNF-α, IL-6, IL-1β, and IL-17A levels, and PAP-1 co-treatment significantly reduced TNF-α and IL-6 levels along with a robust reduction in IL-17A levels (p = 0.057 vs. ODE). In primary microglia **(g)**, ODE increased TNF-α, IL-6, IL-1β, IL-10, IL-17A, and IFNγ levels, with no significant reduction following PAP-1 treatment. Nitrite assay results are expressed in µM and cytokine data is expressed in pg/mL. Sample sizes included n = 3 mouse pups for primary microglia and n = 3 independent cultures for BV2 microglia. Statistical significance is indicated by * for ODE exposure effects (control vs. ODE) and # for PAP-1 treatment effects (ODE vs. ODE + PAP1). *^/#^p < 0.05, **^/##^p < 0.01, ***^/###^p < 0.001, ****^/####^p < 0.0001 by one-way ANOVA with Tukey’s multiple comparisons.

**FIGURE 7:**
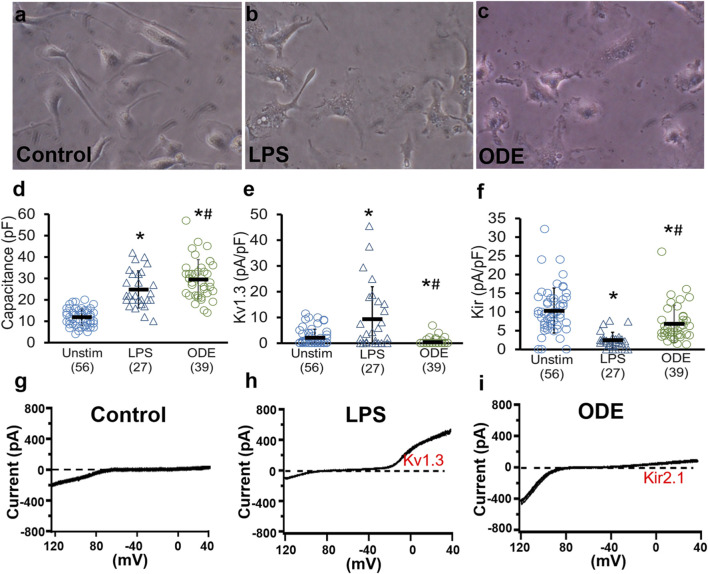
Electrophysiological and morphological changes in primary microglia following LPS and ODE stimulation. Primary microglia were either treated with vehicle (DMEM in 2% FBS), LPS (100 ng/mL), or ODE (1% v/v), and whole-cell patch-clamp recordings were performed at 48 h. Light microscopic microphotographs are shown to visualize morphological changes in microglia **(a–c)**. Kv1.3 and Kir2.1 current density following treatment with DMEM with 2% FBS (control), LPS (100 ng/mL), and ODE (1% w/v) after 48-h are shown as scatterplots from individual cells **(d–f)**. Representative potassium current recordings from control, LPS or ODE treated microglia **(g–i)**. The numbers in parentheses show the number of individual cells that were recorded. * signifies differences compared to the unstimulated condition, and the # signifies differences between the ODE and LPS-treated groups. *^/#^p < 0.05 by unpaired t-test to compare the two groups for statistical significance.

Data were expressed as mean ± SEM and analyzed by one-way ANOVA or two-way ANOVA followed by Tukey’s *post hoc* comparison tests (GraphPad Prism 10.0, La Jolla, California). A p-value of ≤0.05 was considered statistically significant. For Figures 1–6, an asterisk (*) indicates a significant difference between controls vs. ODE only treated cells, whereas a hashtag (#) indicates a significant difference between ODE vs. ODE + PAP-1 treatment. For all BSC, primary microglia, and BV2 experiments, the reported n values represent independent biological replicates (i.e., BSCs derived from different pups, primary microglia isolated from different animals, and independent BV2 culture preparations). For each experiment, measurements were first averaged per animal or per independent culture, and these averaged values were then used for statistical analysis. For Figure 7, * signifies differences compared to the unstimulated condition, and the # signifies differences between the ODE and LPS-treated groups. The details of the statistical analysis applied for each experiment are listed in the corresponding figure legends.

## 3 Results

### 3.1 ODE drives microglial transcriptomic alterations and enhances Kv1.3, NOX2, and p-p38 MAPK signaling in the mouse brain

A comprehensive transcriptomic analysis of microglia isolated from mice exposed to organic dust extract (ODE) identified 19 differentially expressed genes (DEGs), each with a minimum 2-fold change and a p-value ≤0.05. Among these DEGs, 17 were upregulated and 2 downregulated (Figures 1a,b; Supplementary Table 1). Pathway enrichment analysis using DAVID (The Database for Annotation, Visualization, and Integrated Discovery) highlighted significant enrichment predominantly within biological processes (BP) and molecular functions (MF), as visualized by clustering (Figure 1c; Supplementary Table 2). Validation by qPCR was performed on 40 selected genes, including some identified through RNA-seq analysis and others chosen based on their involvement in significantly enriched pathways (Supplementary Table 3). Six genes, Hif1α, Hif2α, E2f2, Ddx3y, Kcna3, and Xylt1, showed consistent and significant validation, exhibiting log_2_ fold-changes ≥2 relative to controls (Figure 1d). Additionally, Western blot analyses after 5 weeks of ODE exposure revealed increased protein expression levels of Kv1.3, NOX2, and phosphorylated-p38 MAPK (p-p38 MAPK) in microglial fraction of mouse brains compared to control groups (Figures 1e–g).

### 3.2 ODE induces kcna3 and MAPK14 expression in BSCs, BV2 microglia and primary microglia

At the end of the study, total RNA (free from genomic DNA) was extracted from BSCs, BV2 microglia, and primary microglia and subsequently analyzed using qPCR (Figure 2). The results revealed a significant increase in the gene expression of kcna3 (Figures 2a–c) and MAPK14 (Figures 2d–f) in BSCs, BV2 microglia, and primary microglia compared to the control group. Notably, treatment with PAP-1 significantly reduced the expression levels of kcna3 (Figures 2a–c) and MAPK14 (Figures 2d–f) in ODE-treated BSCs, BV2 microglia, and primary microglia.

### 3.3 ODE induces Kv1.3 expression in BSCs, BV2 microglia and primary microglia

Following treatments with ODE and PAP-1, BSCs, BV2 microglia, and primary microglia were analyzed for cellular expression of Kv1.3 protein using immunostaining (Figure 3). In BSCs, co-staining with Iba1 was performed to specifically identify microglia and confirm that Kv1.3 expression was quantified exclusively within microglial cells; representative Iba1 staining and colocalization images are provided in Supplementary Figure 1. Quantitative analysis of fluorescent images in BSCs (Figures 3a,b), BV2 microglia (Figures 3c,d), and primary microglia (Figures 3e,f) revealed mean Kv1.3 expression per cell. ODE exposure resulted in a significant increase in Kv1.3 protein expression in BSCs (Figure 3b), BV2 microglia (Figure 3d), and primary microglia (Figure 3f) compared to the control group. However, co-treatment with PAP-1 significantly reduced ODE-induced Kv1.3 protein expression in BSCs (Figures 3a,b), BV2 microglia (Figures 3c,d), and primary microglia (Figures 3e,f).

### 3.4 ODE induces NOX2 expression in BSCs and primary microglia

Following treatments with ODE and PAP-1, BSCs and primary microglia were analyzed for cellular expression of NOX2 protein using immunostaining (Figure 4). In BSCs, co-staining with Iba1 was performed to specifically identify microglia and confirm that NOX2 expression was quantified exclusively within microglial cells; representative Iba1 staining and colocalization images are provided in Supplementary Figure 2. Quantitative analysis of fluorescent images revealed mean NOX2 expression per cell in BSCs (Figures 4a,b) and primary microglia (Figures 4c,d). ODE exposure resulted in a significant increase in NOX2 protein expression in BSCs (Figure 4b) and primary microglia (Figure 4d) compared to the control group. However, co-treatment with PAP-1 significantly reduced ODE-induced NOX2 protein expression in BSCs (Figures 4a,b) and primary microglia (Figures 4c,d).

### 3.5 ODE induces p-p38 MAPK expression in BSCs, BV2 microglia, and primary microglia

Following treatments with ODE and PAP-1, BSCs, BV2 microglia, and primary microglia were analyzed for cellular expression of p-p38 MAPK protein using immunostaining (Figure 5). In BSCs, co-staining with Iba1 was performed to specifically identify microglia and confirm that p-p38 MAPK expression was quantified exclusively within microglial cells; representative Iba1 staining and colocalization images are provided in Supplementary Figure 3. Quantitative analysis of fluorescent images in BSCs (Figures 5a,b), BV2 microglia (Figures 5c,d), and primary microglia (Figures 5e,f) revealed mean p-p38 MAPK expression per cell. ODE exposure resulted in a significant increase in p-p38 MAPK protein expression in BSCs (Figure 5b), BV2 microglia (Figure 5d), and primary microglia (Fig. 5f) compared to the control group. However, co-treatment with PAP-1 significantly reduced ODE-induced p-p38 MAPK protein expression in BSCs (Figures 5a,b), BV2 microglia (Figures 5c,d), and primary microglia (Figures 5e,f).

### 3.6 ODE exposure induces nitrite production and pro-inflammatory cytokine secretion in brain slice cultures, BV2, and primary microglia

Cell culture supernatants from BSCs, BV2 microglia, and primary microglia were analyzed following ODE exposure. Nitrite secretion significantly increased in supernatants from BSCs, BV2 microglia, and primary microglia compared to controls, while co-treatment with PAP-1 markedly reduced nitrite levels across all cell types (Figures 6a–c). Additionally, pro-inflammatory cytokine levels were assessed using ELISA for BSCs and multiplex assay for BV2 and primary microglia. In BSCs, ODE exposure significantly elevated the secretion of TNF-α and IL-6, with PAP-1 treatment effectively reducing IL-6 but not TNF-α levels (Figures 6d,e). Similarly, multiplex assays revealed significantly increased secretion of TNF-α, IL-6, IL-1β, and IL-17A in BV2 microglia (Figure 6f), and elevated levels of TNF-α, IL-6, IL-1β, IL-10, IL-17A, and IFNγ in primary microglia (Figure 6g). PAP-1 co-treatment notably decreased TNF-α, IL-6, secretion and showed a robust reduction in elevated IL-17A levels with p = 0.057induced by ODE exposure in BV2 microglia but had no significant effect on other cytokines tested (Figure 6f).

### 3.7 ODE exposure increases electrical capacitance without affecting Kir2.1 and Kv1.3 currents in primary microglia

Primary microglia were treated with either medium as a negative control (DMEM with 2% FBS), LPS (100 ng/mL) as a positive control, or ODE (1% v/v) for 48 h. Whole-cell patch-clamp recordings with ramp pulses which allow us to detect both Kir currents below −80 mV and Kv1.3 currents above −40 mV were performed on individual microglia at 48 h following treatment. Light microscopic evaluation revealed a notable increase in microglial cell size with an ameboid morphology after LPS and ODE stimulation compared to unstimulated controls (Figures 7a–c), indicating an activated state. Interestingly, while LPS induced Kv1.3 currents and decreased Kir2.1 currents as expected, organic dust did not induce significant Kv1.3 currents on the plasma membrane or decrease Kir2.1 currents (Figures 7d–i). ODE certainly activated microglia based on the cell shape and the increase in electrical cell capacitance, which indicates a roughly 3-fold increase in cell surface area following ODE exposure (Figure 7d).

## 4 Discussion

Agricultural regions in the United States have been associated with exposure to agricultural contaminants that may play a role in initiating and perpetuating neuroinflammatory cycles, ultimately contributing to neurodegeneration (Wright Willis et al., 2010; Arora et al., 2021). Building on previous work (Massey et al., 2019; Massey et al., 2021; Massey et al., 2022), we investigated how inhaled ODE induces sustained neurotoxic signaling. Chronic neuroinflammation involves complex interactions among brain and peripheral immune cells *via* cytokines, chemokines, ROS, and damage-associated molecular patterns (DAMPs). Transcriptomic analysis can unravel complex pathways and help identify specific therapeutic targets.

Our current findings provide new insights into the transcriptomic and molecular pathways underlying ODE-induced neuroinflammation and neurodegeneration (Massey et al., 2022). ODE exposure significantly upregulates the expression of the *kcna3* gene, which encodes the Kv1.3 potassium channel. Kv1.3 is pivotal in microglial activation and the subsequent neuroinflammatory response (Rangaraju et al., 2017). Upregulation of Kv1.3 in microglia has been observed in Alzheimer’s and Parkinson’s diseases (Fordyce et al., 2005; Maezawa et al., 2018; Sarkar et al., 2020). Upregulation of Kv1.3 promotes pro-inflammatory cytokine and ROS production, exacerbating neuronal damage. Inhibiting Kv1.3 can reduce microglial activation and neuroinflammation, making it a promising drug target. Studying the *kcna3* gene may reveal links between gene expression and cellular dysfunctions, offering insights into microglial pathology and neurotoxicity (Sarkar et al., 2020).

In this study, the upregulation of *kcna3* mRNA was accompanied by increased *mapk14* mRNA which encodes for p38α MAPK, a kinase playing a central role in cellular responses to stress and inflammation, by regulating immune responses, apoptosis, and cytokine production (Canovas and Nebreda, 2021). Activation of p38 MAPK produces pro-inflammatory mediators, contributing to neurotoxicity, while the selective targeting can reduce neuroinflammation (Gee et al., 2020). The interaction between Kv1.3 channels and the p38 MAPK pathway forms a signaling loop to activate microglia and increase neuroinflammation.

Targeted inhibition of this signaling loop could offer a therapeutic strategy to reduce neuroinflammation (Rangaraju et al., 2017). This signaling cascade has been shown to modulate Kv1.3 levels via Fyn Kinase-mediated p38 MAPK phosphorylation and NF-κB pathway activation (Panicker et al., 2015; Sarkar et al., 2020). Additionally, p-p38 MAPK enhances microglial neurotoxicity by promoting Kv1.3 expression and the generation of peroxynitrite, a neurotoxic compound formed from the interaction of ROS and nitrite (Liu et al., 2012; Fordyce et al., 2005; Torreilles et al., 1999). In our study, brain tissues from mice exposed to ODE for 5 weeks demonstrated significant increases in Kv1.3, p-p38 MAPK, and NOX2, implicating oxidative stress as a key driver of neuroinflammatory signaling. To assess the translational relevance of our findings, we utilized three experimental models: BSCs, immortalized BV2 microglial cell line, and primary microglia, where a significant elevation in *kcna3 mRNA* and *mapk14* mRNA was observed post-ODE exposure. Increased levels of microglial Kv1.3 and phosphorylated p38 MAPK were confirmed across all models.

Selective blockers of Kv1.3, such as PAP-1 and ShK-223, can reduce microglial activation and production of pro-inflammatory cytokines, and could be developed as therapeutic agents for reducing neuroinflammation (Rangaraju et al., 2017). In the ODE exposure models, Kv1.3 inhibition with PAP-1 resulted in a significant reduction in the mRNA of *kcna3* and *mapk14,* along with Kv1.3 and p-p38 MAPK protein levels. Interestingly, p38α MAPK inhibitors have also been shown to reduce neuropathology and improve cognitive function. In an alternate strategy, dual targeting of Kv1.3 channels and the p38 MAPK pathway could also represent a powerful approach for controlling microglial activation and mitigating neuroinflammation (Garrett and Kyriacou, 1988).

Activated microglia produce excess ROS via NOX2, which damages cells, including neurons, and exacerbates inflammation (Houldsworth, 2024). Oxidative stress also activates the MAPK pathway, triggering pro-inflammatory cytokine release, leading to a self-perpetuating loop of inflammation. Elevated p38 MAPK and oxidative stress markers are observed in Alzheimer’s and Parkinson’s brains (Bernhardi, 2009). We validated this relationship between Kv1.3 and oxidative stress in our model by examining the expression of NOX2. Consistent with our previous findings in an immortalized microglial cell line (Massey et al., 2019), ODE exposure significantly increased the NOX2 protein expression in primary microglia and BSCs. Elevated levels of Kv1.3 and p-p38 MAPK suggest that Kv1.3 channel activity contributes to oxidative stress**,** promoting the formation of peroxynitrite**,** a neurotoxic compound linked to neuronal damage**.** PAP-1 effectively reduces these effects, reinforcing Kv1.3’s role both as a biomarker for microglial activation and a therapeutic target in neuroinflammation, particularly in the ODE exposure model**.** Further research into the molecular mechanisms linking Kv1.3 and oxidative stress could lead to better treatment for neuroinflammatory conditions (Jembrek, 2024).

Our efforts to resolve the inflammatory milieu showed that ODE exposure significantly increased the release of TNF-α, IL-6, IL-1β, and IL-17A, as previously shown. In primary microglial cultures, a broader spectrum of cytokines, including IL-10 and IFNγ, was observed, indicative of robust microglial activation and engagement of both pro- and anti-inflammatory pathways. PAP-1 treatment selectively reduced IL-6 levels but not TNF-α in BSCs, while the presence of IL-10 suggests concurrent activation of anti-inflammatory signaling. In contrast, in the BV2 microglial model, PAP-1 reduced both TNF-α and IL-6 without significantly affecting other cytokines. Notably, although PAP-1 attenuated multiple inflammatory and oxidative stress markers, its effects on cytokine production were not uniform across all models, particularly in primary microglia where TNF-α was not consistently reduced. This variability suggests that Kv1.3 inhibition selectively modulates specific inflammatory pathways rather than acting as a global suppressor of cytokine release. These differential responses likely reflect intrinsic differences among experimental systems. BSCs comprise multiple brain cell types and preserve neuron–glia interactions, whereas BV2 microglia are immortalized and primary microglia are isolated systems lacking paracrine neuronal and astrocytic signaling. In addition, differences in exposure duration (5 days in BSCs versus 48 h in BV2 and primary microglial cultures), along with developmental and activation-state differences, may further influence cytokine dynamics and responsiveness to Kv1.3 inhibition.

Despite these differences, Kv1.3 inhibition consistently reduced nitrite production, NOX2 expression, and p38 MAPK phosphorylation across all models, demonstrating that Kv1.3 signaling lies upstream of the oxidative stress pathways activated by ODE. Because oxidative stress is a well-recognized trigger of pro-inflammatory cascades via MAPK and NF-κB signaling, these findings align with prior reports that Kv1.3 blockade mitigates oxidative stress–driven inflammation in models of multiple sclerosis, Alzheimer’s disease, and traumatic brain injury (Angi et al., 2025; Markakis et al., 2021; Mizuma et al., 2019; Rangaraju et al., 2009; Revuelta et al., 2022; Sarkar et al., 2020). The lack of consistent cytokine suppression despite reduced oxidative markers suggests that Kv1.3 primarily regulates early redox-sensitive signaling rather than directly modulating downstream cytokine release, which may also vary with timing and cellular context. Overall, our data establishes a causal link between Kv1.3 activity and ODE-induced oxidative stress, positioning Kv1.3 as an upstream control point in the neuroinflammatory cascade. Future studies using *in vivo* mouse models, concentration–response and time-course analyses, and Kv1.3 knockdown approaches will help further delineate these mechanistic pathways, including the potential roles of TLRs, MAPKs, and inflammasomes.

Whole-cell patch-clamp recordings revealed no increase in plasma membrane Kv1.3 currents in ODE-stimulated primary microglia despite elevated intracellular Kv1.3 protein levels. This dissociation indicates that increased Kv1.3 expression does not necessarily translate into enhanced surface conductance and suggests altered channel localization under ODE exposure. While not directly tested here, this pattern is consistent with disrupted protein trafficking, potentially arising from ER and mitochondrial dysfunction, processes previously shown by our group to be impaired following ODE exposure (Massey et al., 2021; Bowen et al., 2024).

In this context, ER stress previously observed in our ODE models (Massey et al., 2021), may plausibly interfere with Kv1.3 protein folding, processing, or export, limiting its delivery to the plasma membrane and promoting intracellular retention within the ER or other compartments. In addition, Kv1.3 is known to localize to mitochondria (mitoKv1.3), where it regulates mitochondrial membrane potential, reactive oxygen species (ROS) production, and apoptotic signaling. Dysregulation at this level could contribute to mitochondrial dysfunction and amplification of oxidative and pro-inflammatory signaling, even in the absence of increased surface currents (Capera et al., 2022; Wang et al., 2022).

Despite the absence of enhanced surface Kv1.3 currents, we observed a significant increase in pro-inflammatory cytokine release, elevated NOX2 protein expression, and nitrite production following ODE exposure. These outcomes are consistent with the hypothesis that intracellular Kv1.3, either in the ER or mitochondria, remains functionally active and contributes to these inflammatory pathways (Checchetto et al., 2019; Fukai and Ushio-Fukai, 2020; Sarkar et al., 2020). Specifically, ER-localized Kv1.3 (ERKv1.3) may alter calcium signaling and protein folding homeostasis, exacerbating ER stress responses and downstream cytokine release (Pontisso et al., 2024). Inhibition of ER stress pathways and restoration of mitochondrial function could thus represent promising therapeutic avenues, not only to address Kv1.3 trafficking defects but also to mitigate the broader inflammatory and oxidative stress responses triggered by ODE exposure.

The molecular complexity of ODE may further contribute to altered Kv1.3 regulation. ODE exposure likely activates multiple signaling pathways, including Fyn kinase and NF-κB signaling, which could indirectly influence Kv1.3 expression, localization, or turnover. In addition, while transcriptomic profiling was performed using microglia isolated from adult mice following *in vivo* ODE exposure, mechanistic validation experiments, including electrophysiological recordings, were conducted using neonatal primary microglia, BV2 cells, and *ex vivo* brain slice cultures. Given well-established developmental differences in microglial maturation, ion channel regulation, and membrane properties, this distinction may contribute to the observed lack of increased plasma membrane Kv1.3 currents in ODE-stimulated neonatal primary microglia despite elevated intracellular Kv1.3 protein levels. Together, these considerations suggest that temporal and developmental regulation of Kv1.3 trafficking and function may be critical, and future studies should assess Kv1.3 localization and electrophysiological activity across developmental stages and multiple time points following ODE exposure. From a therapeutic perspective, pharmacological inhibition of Kv1.3 with PAP-1 attenuated neuroinflammatory markers in this study; however, whether Kv1.3 inhibition directly influences channel trafficking or intracellular localization remains unresolved. Future investigations employing surface biotinylation, super-resolution microscopy, live-cell imaging, and Kv1.3-specific genetic knockdown approaches will be necessary to directly test these mechanisms.

Our study measured NOX2 and nitrite levels via Griess assay or ICC, whereas the use of either N-Tyr or fluorescent assays would have provided robust confirmation of the oxidative stress. Another limitation is the absence of normalization of phosphorylated p38 MAPK (p-p38 MAPK) to total p38 MAPK in the Western blot analysis. Ideally, normalization to total protein levels provides a more precise understanding of activation-specific effects. However, actin levels were consistent between control and treatment groups within each experiment (Figure 1). This within-experiment stability supports the use of β-actin as a valid loading control for assessing relative changes in p-p38 MAPK induced by ODE treatment. Our primary goal was to determine whether there is a general upregulation of the phosphorylated (active) form of p38 MAPK, serving as an indicator of pathway activation, and to further test these findings in *in vitro* and *ex vivo* models.

**FIGURE 8:**
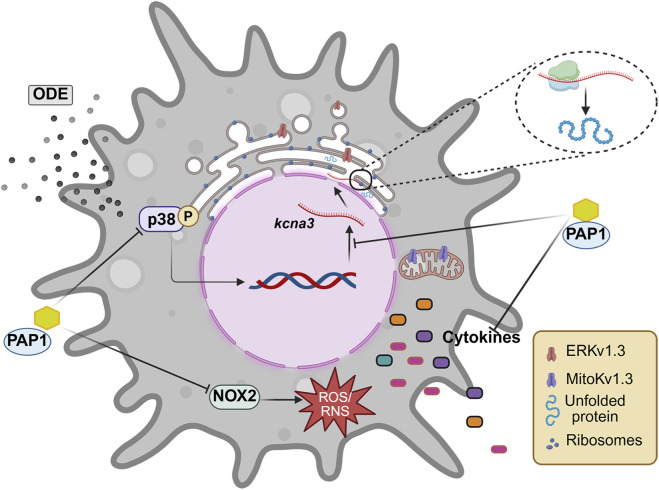
An overview of ODE exposure-induced kcna3 upregulation in primary microglia. ODE exposure mediated an increase in p38 MAPK phosphorylation, leading to upregulated *kcna3* expression. This was accompanied by elevated Kv1.3 protein levels in microglia following ODE stimulation. Additionally, ODE exposure triggered the secretion of pro-inflammatory cytokines and nitrite. Treatment with PAP-1 effectively reduced Kv1.3 protein levels, p38 MAPK phosphorylation, and nitrooxidative stress markers. However, PAP-1 treatment did not significantly alter pro-inflammatory cytokine secretion in response to ODE.

This study increases our understanding of Kv1.3’s complex role in ODE-induced neuroinflammation. Although intracellular Kv1.3 upregulation contributes to oxidative stress and cytokine release, its absence on the cell surface and lack of associated currents suggest intricate regulatory mechanisms. The findings highlight Kv1.3 as a promising therapeutic target. Future research should examine Kv1.3 trafficking, the timing of oxidative and inflammatory responses, and other pathways involved in microglial activation to develop targeted treatments for neurodegenerative diseases (Figure 8).

## Data Availability

The original contributions presented in the study are included in the article/Supplementary Material, further inquiries can be directed to the corresponding author.
