# Supplementary material for: Kv1.3 as an upstream regulator of oxidative stress-mediated neuroinflammation following organic dust exposure in murine in vitro, ex vivo, and in vivo models
Source: Front Toxicol. 2026 Mar 24;8:1765108. doi: 10.3389/ftox.2026.1765108 (PMC13053031; doi:10.3389/ftox.2026.1765108)
Supplement: Supplementary file 1 [file DataSheet1.pdf]

Supplementary Material

Kv1.3 as an Upstream Regulator of Oxidative Stress–Mediated Neuroinflammation Following Organic Dust Exposure in Murine In Vitro, Ex Vivo, and In Vivo Models

Nyzil Massey[1¥], Sanjana Mahadev Bhat[2], Denusha Shrestha[3], Emir Malovic[4], Locke A. Karriker[5], Shivani Choudhary[1], Alan P. Robertson[1], Hai Minh Nguyen[6], Heike Wulff[6], Anumantha G. Kanthasamy[7], and Chandrashekhar Charavaryamath[8]

[1]Biomedical Sciences, Iowa State University, Ames, IA, 50011

[2]Mayo Clinic, Rochester, MN, 55905

[3]South Dakota State University, Brookings, SD, 57007

[4]University of Illinois Chicago, Chicago, IL, 60607

[5]VDPAM, Iowa State University, Ames, IA, 50011

[6]Department of Pharmacology, School of Medicine, University of California, Davis, CA 95616

[7]Isakson Center for Neurological Disease Research, Department of Physiology and Pharmacology, College of Veterinary Medicine, University of Georgia, Athens, GA, 30602

[8]Department of Veterinary Biomedical Sciences, Shreiber School of Veterinary Medicine (SSVM), Rowan University, Glassboro, NJ, 08062

¥Correspondence:
Corresponding author
nyzil@iastate.edu

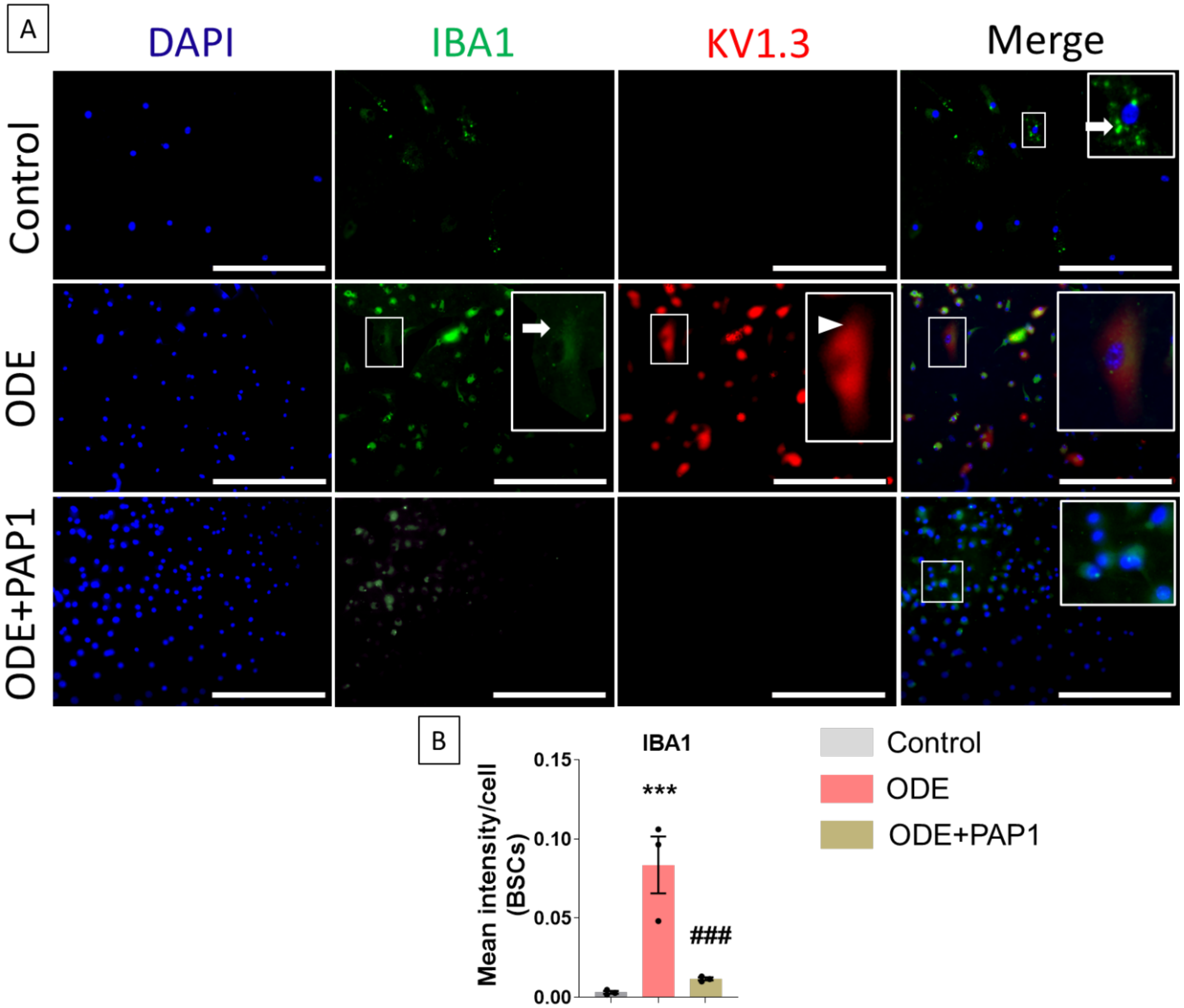

Supplementary Figure 1 ODE induces IBA1 and KV1.3 expression in BSCs. Treated (Table 13.1) BSCs were fixed with 4% paraformaldehyde. Following fixing, BSCs were co-stained with anti IBA1 (Fitc, green) antibody and Kv1.3 (Cy3, red). IBA1 and Kv1.3 expression in IHC was quantified, compared to control, ODE-exposed mice showed higher increased IBA1 staining intensity. PAP1 treatment significantly reduced IBA1 expression (n=3,* exposure effect, # PAP1 treatment effect, $p \leq 0.05$, micrometer bar = 50 µm).

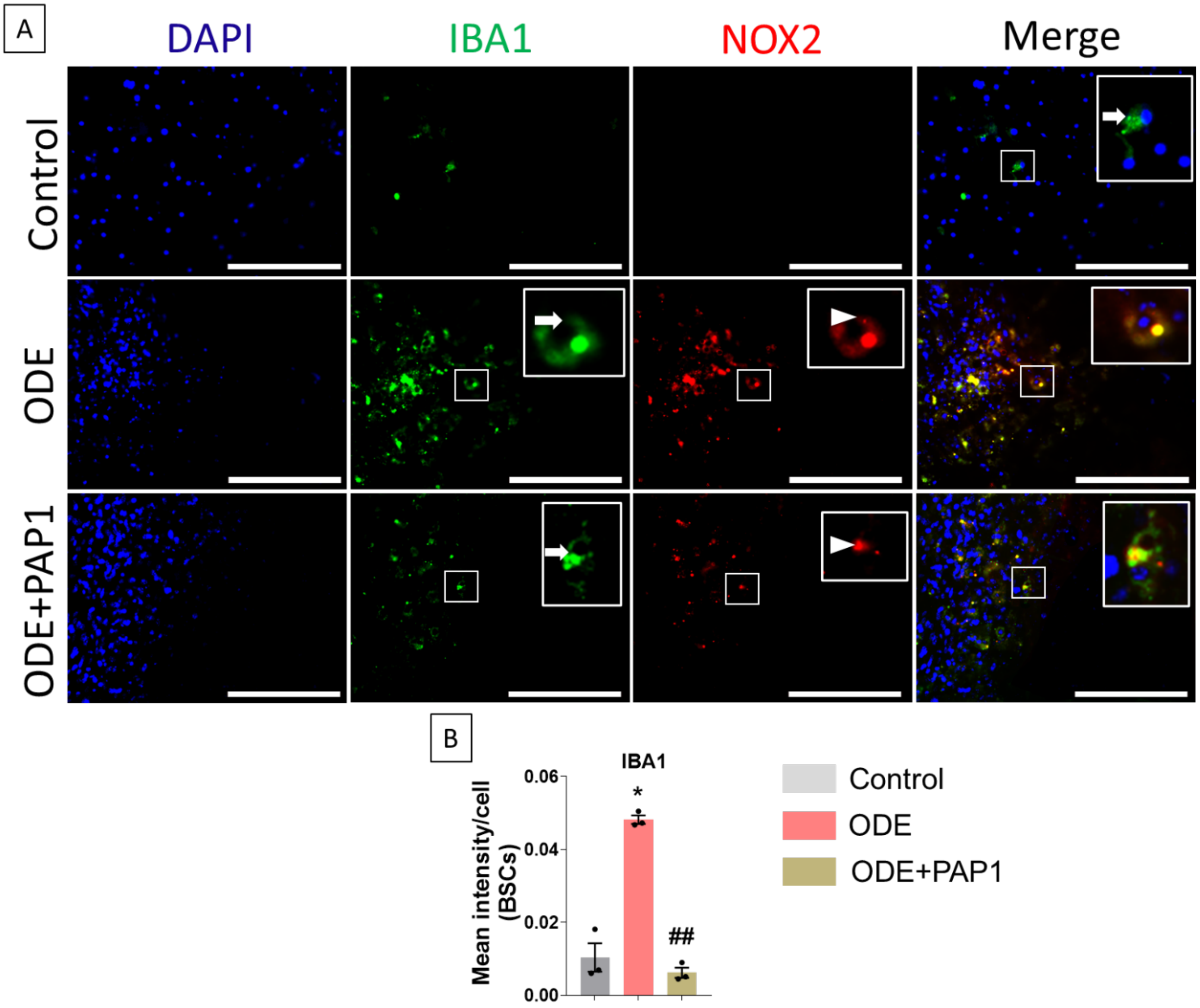

Supplementary Figure 2 ODE induces IBA1 and NOX2 expression in BSCs. Treated (Table 13.1) BSCs were fixed with 4% paraformaldehyde. Following fixing, BSCs were co-stained with anti IBA1 (Fitc, green) antibody and NOX2 (Cy3, red). IBA1 and NOX2 expression in IHC was quantified, compared to control, ODE-exposed mice showed higher increased IBA1 staining intensity. PAP1 treatment significantly reduced IBA1 expression (n=3,* exposure effect, # PAP1 treatment effect, $p \leq 0.05$, micrometer bar = 50 μm).

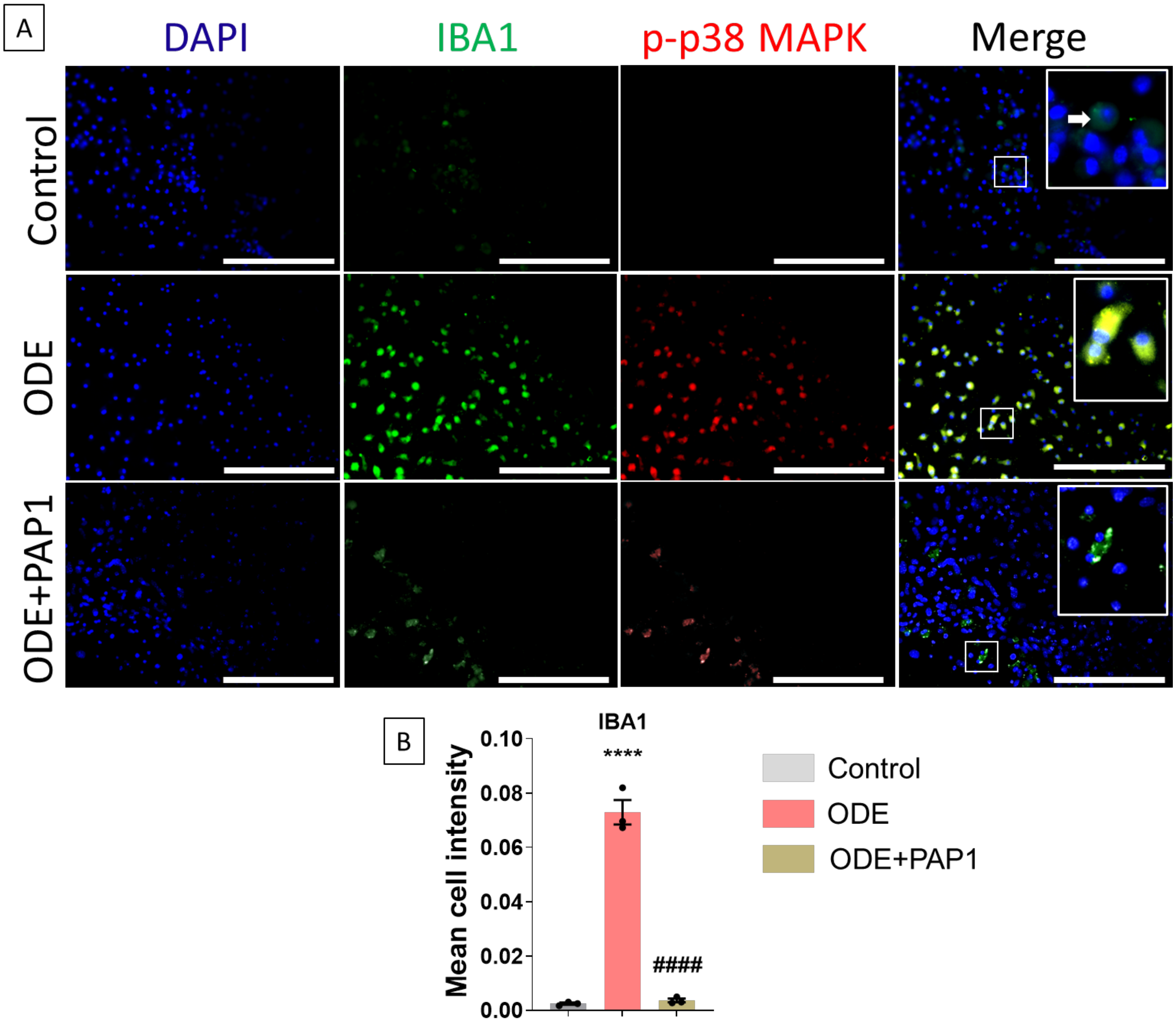

Supplementary Figure 3 ODE induces IBA1 and p-p38 MAPK expression in BSCs. Treated (Table 13.1) BSCs were fixed with 4% paraformaldehyde. Following fixing, BSCs were co-stained with anti IBA1 (Fitc, green) antibody and p-p38 MAPK (Cy3, red). IBA1 and p-p38 MAPK expression in IHC was quantified, compared to control, ODE-exposed mice showed higher increased IBA1 staining intensity. PAP1 treatment significantly reduced IBA1 expression (n=3,* exposure effect, # PAP1 treatment effect, $p \leq 0.05$, micrometer bar = 50 µm).

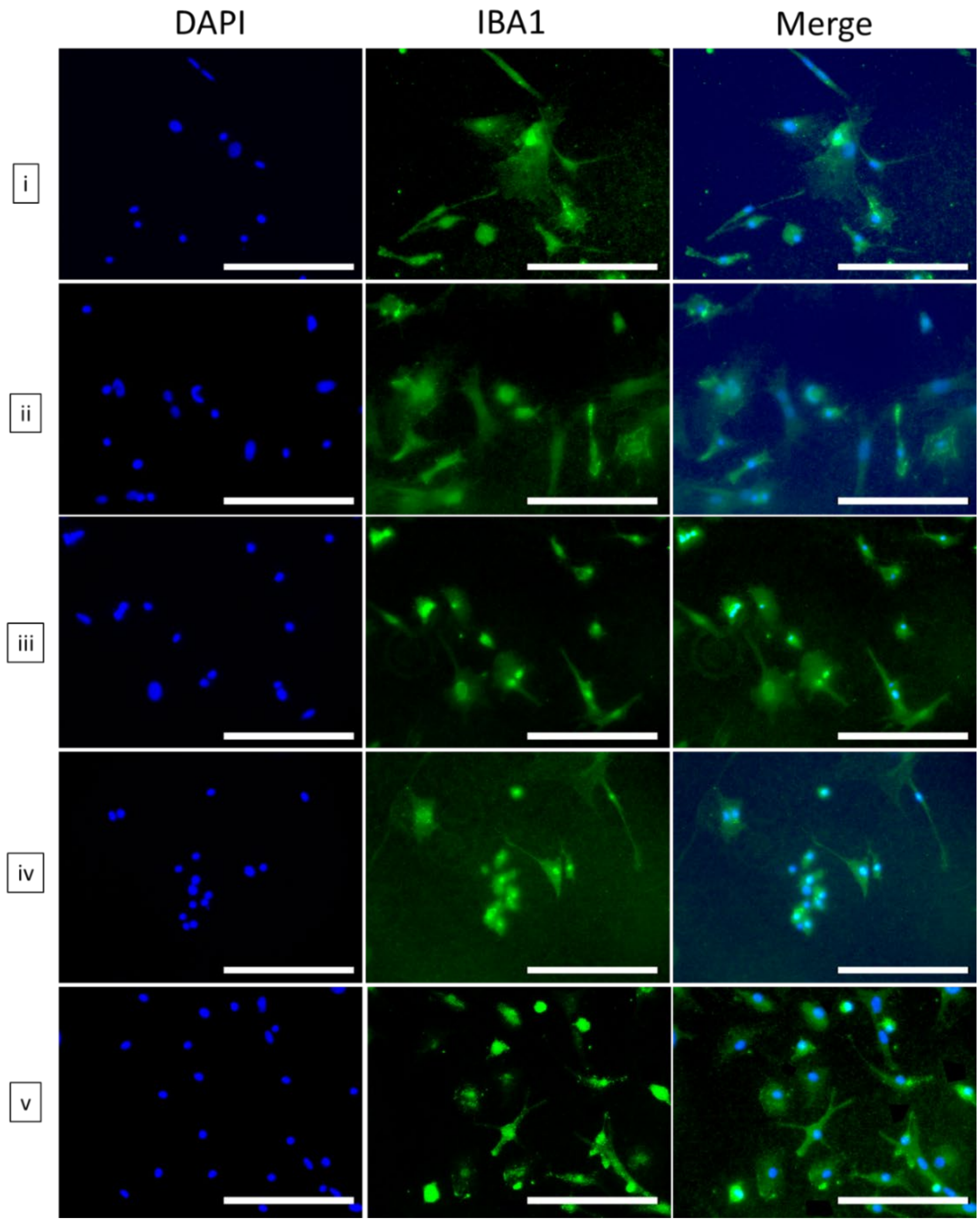

Number of images	Total cells	IBA1 positive cells	IBA1 positive (percent)
1	13	12	92.30769231
2	17	16	94.11764706
3	15	15	100
4	14	14	100
5	18	17	94.44444444
	Cumulative percentage		96.17395676

Supplementary Figure 4. IBA1-positive cells following magnetic isolation. Following magnetic isolation of microglia from mixed glial culture, magnetically isolated cells were stained with anti-IBA1 (FITC) staining and counted. More than 96% of cells per field expressed IBA1, indicating a highly purified fraction.

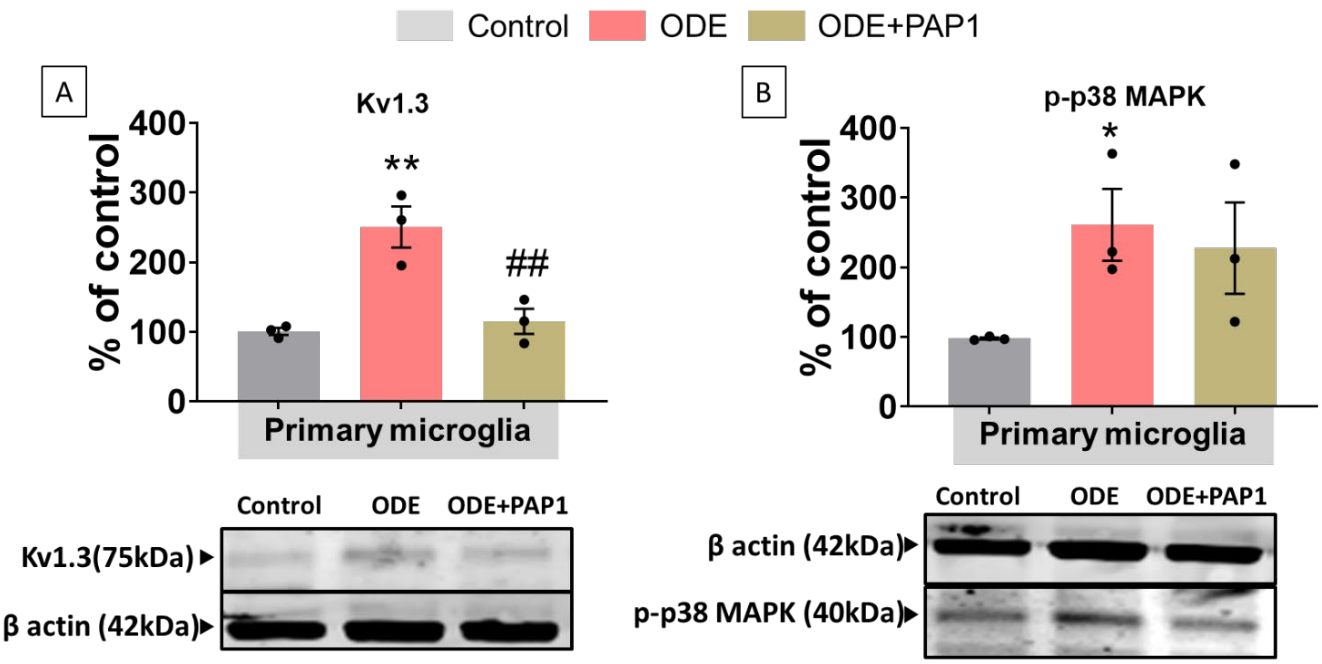

Supplementary Figure 5. ODE induces Kv1.3 and p-p38 MAPK protein expression in primary microglia. Whole cell lysate from WTMCL were processed for western blot analysis. KV 1.3, p-p38 MAPK and β-actin antibodies (house-keeping protein) detected 75 kDa, 40kDa and 42 kDa bands, respectively. Densitometry of normalized bands showed that, compared to controls, ODE exposure increased the Kv1.3 (A) and p-p38 MAPK (B) protein levels. PAP1 treatment significantly reduced both Kv1.3 (A) and p-p38 MAPK (B) protein level (n=3, * exposure effect, # PAP1 treatment effect, $p \leq 0.05$).

A. Kv1.3

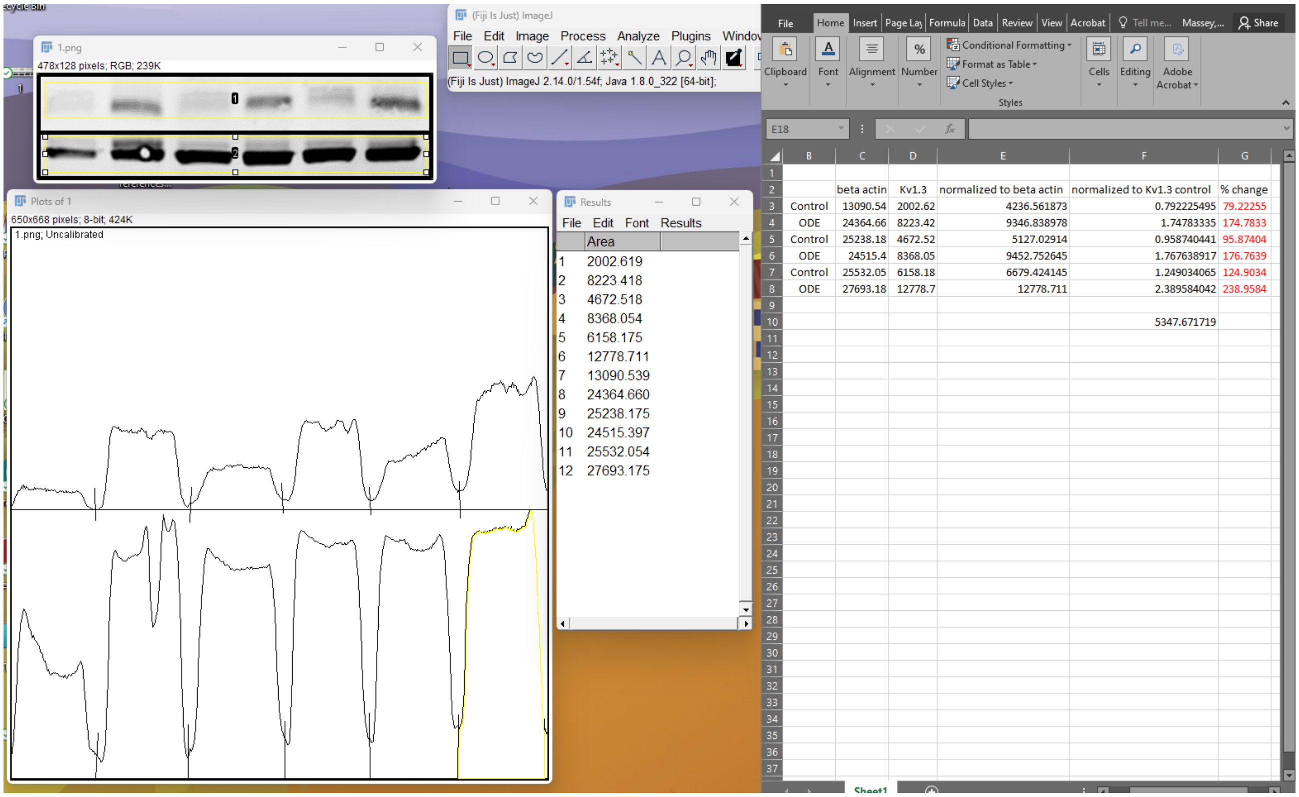
1.png
478x128 pixels; RGB; 239K
(Fiji Is Just) ImageJ
File Edit Image Process Analyze Plugins Window
(Fiji Is Just) ImageJ 2.14.0/1.54f; Java 1.8.0_322 [64-bit];
Plots of 1
650x668 pixels; 8-bit; 424K
1.png; Uncalibrated
Results
File Edit Font Results
Area
1 2002.619
2 8223.418
3 4672.518
4 8368.054
5 6158.175
6 12778.711
7 13090.539
8 24364.660
9 25238.175
10 24515.397
11 25532.054
12 27693.175
File Home Insert Page Lay Formula Data Review View Acrobat Tell me... Massey,... Share
Conditional Formatting
Format as Table
Cell Styles
Clipboard Font Alignment Number Styles Cells Editing Adobe Acrobat
E18
beta actin Kv1.3 normalized to beta actin normalized to Kv1.3 control % change
Control 13090.54 2002.62 4236.561873 0.792225495 79.22255
ODE 24364.66 8223.42 9346.838978 1.74783335 174.7833
Control 25238.18 4672.52 5127.02914 0.958740441 95.87404
ODE 24515.4 8368.05 9452.752645 1.767638917 176.7639
Control 25532.05 6158.18 6679.424145 1.249034065 124.9034
ODE 27693.18 12778.7 12778.711 2.389584042 238.9584
5347.671719
Sheet1

B. NOX2

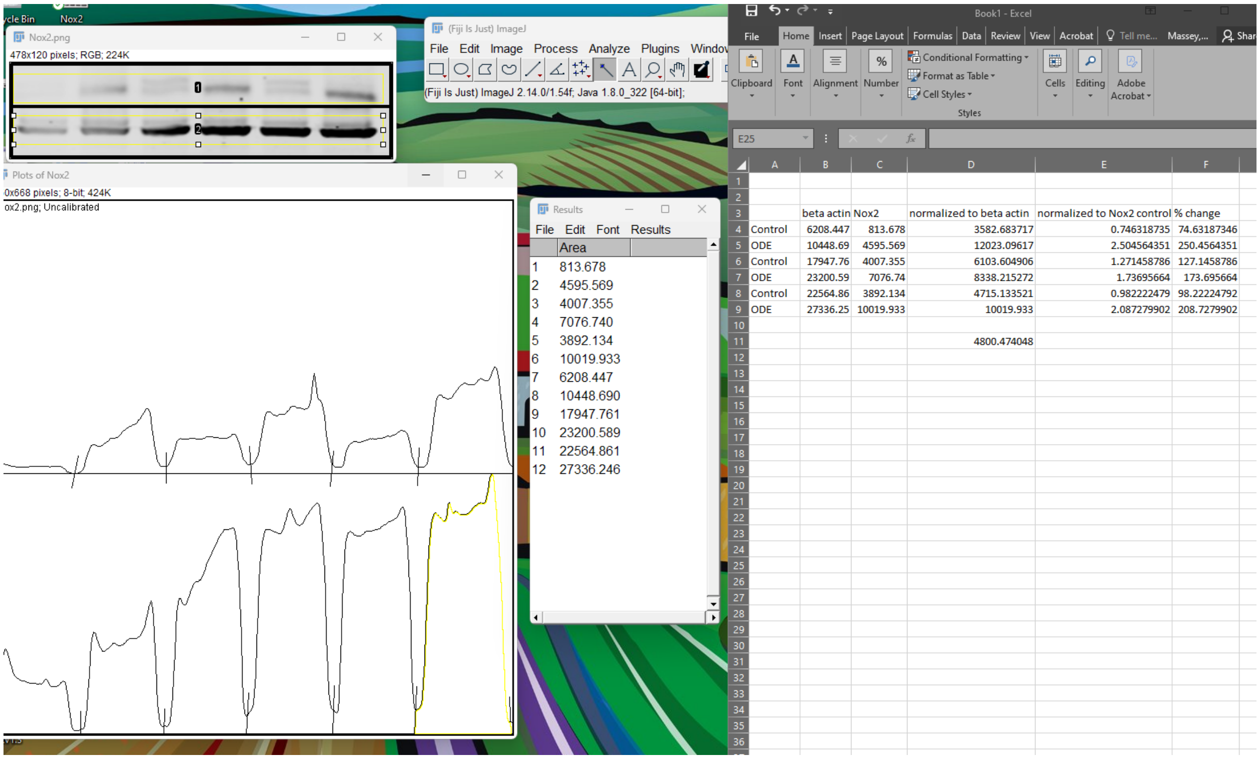
Nox2
Nox2.png
478x120 pixels; RGB; 224K
(Fiji Is Just) ImageJ
File Edit Image Process Analyze Plugins Window
(Fiji Is Just) ImageJ 2.14.0/1.54f; Java 1.8.0_322 [64-bit];
Plots of Nox2
0x668 pixels; 8-bit; 424K
ox2.png; Uncalibrated
Results
File Edit Font Results
Area
1 813.678
2 4595.569
3 4007.355
4 7076.740
5 3892.134
6 10019.933
7 6208.447
8 10448.690
9 17947.761
10 23200.589
11 22564.861
12 27336.246
Book1 - Excel
File Home Insert Page Layout Formulas Data Review View Acrobat Tell me... Massey,...
Conditional Formatting
Format as Table
Cell Styles
Clipboard Font Alignment Number Styles Cells Editing Adobe Acrobat
E25
beta actin Nox2 normalized to beta actin normalized to Nox2 control % change
Control 6208.447 813.678 3582.683717 0.746318735 74.63187346
ODE 10448.69 4595.569 12023.09617 2.504564351 250.4564351
Control 17947.76 4007.355 6103.604906 1.271458786 127.1458786
ODE 23200.59 7076.74 8338.215272 1.73695664 173.695664
Control 22564.86 3892.134 4715.133521 0.982222479 98.22224792
ODE 27336.25 10019.933 10019.933 2.087279902 208.7279902
4800.474048

C. p-p38 MAPK

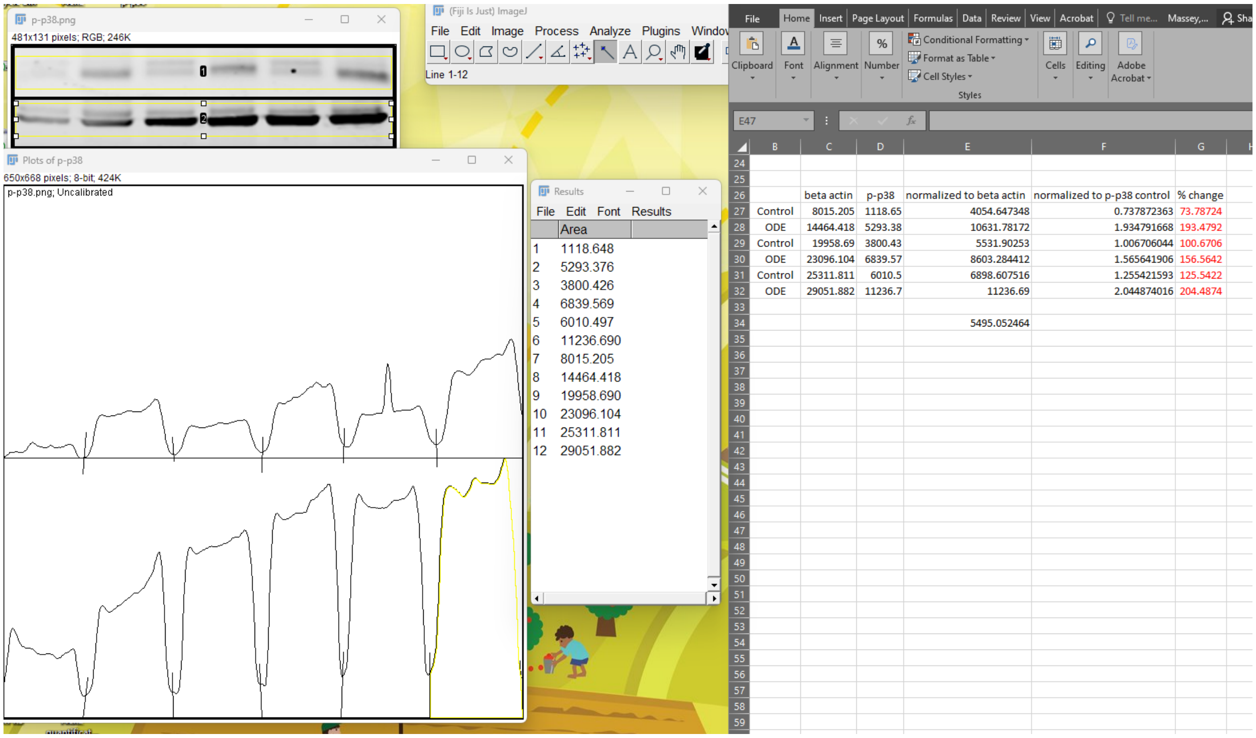

Supplementary figure 6: Western blot quantification in ImageJ of Kv1.3, NOX2 and p-p38 MAPK in isolated microglia from the mouse brain

Supplementary Table 1. DEGs (Control Vs ODE)

Gene symbol	Gene name	Fold change	-log10(p value)
Ddx3y	DEAD (Asp-Glu-Ala-Asp) box polypeptide 3, Y-linked	9.28778	9.6392
Kdm5d	lysine (K)-specific demethylase 5D	8.37495	9.6675
Eif2s3y	eukaryotic translation initiation factor 2, subunit 3, structural gene Y-linked	7.56009	7.99237
Uty	ubiquitously transcribed tetratricopeptide repeat gene, Y chromosome	6.4943	7.3404
Mir3960	microRNA 3960	3.0608	2.97837
Kcna3	potassium voltage-gated channel, shaker-related subfamily, member 3	2.97393	3.2481719
Pth2	parathyroid hormone 2	2.92944	2.72195

Xylt1	xylosyltransferase 1	2.84987	2.078611
E2f2	E2F transcription factor 2	2.72761	2.512473
Abcc6	ATP-binding cassette, sub-family C (CFTR/MRP), member 6	2.70606	2.02487
Ly6g	lymphocyte antigen 6 complex, locus G	2.55231	2.04767
Hif1a	hypoxia-inducible factor 1, alpha subunit inhibitor	2.53759	2.0941934
Gm6878	predicted gene 6878	2.50622	2.62555
Rab44	RAB44, member RAS oncogene family	2.23999	1.47816
Slpi	secretory leukocyte peptidase inhibitor	2.19406	2.79084
Gm5237	predicted gene 5237	2.13247	1.92013
Tstd1	thiosulfate sulfurtransferase (rhodanese)-like domain containing 1	2.06113	3.0008
Erdr1	erythroid differentiation regulator 1	-2.62243	2.57297
Xist	inactive X specific transcripts	-11.0481	11.0693

Supplementary Table 2. Gene ontology analysis of DEGs (Control-ODE)

Enriched pathway	Fold change	Category
positive regulation of transcription from RNA polymerase II promoter	4.2	BP
inflammatory response	3.3	BP
positive regulation of transcription from RNA polymerase II promoter	3.2	BP
positive regulation of apoptotic process	2.5	BP
negative regulation of transcription from RNA polymerase II promoter	2.5	BP
oxidation-reduction process	2.4	BP
signal transduction	2.1	BP
signal transducer activity	2.3	MF
oxidoreductase activity	2	MF

Supplementary Table 3. Primer sequences for validation of mRNA levels by qRT-PCR

Gene	Sequence 5'-3'	Direction
IL-1β	CGCAGCAGCACATCAACAAGAGC	Forward
	TGTCCTCATCCTGGAAGGTCCACG	Reverse
KRT2	GCCTCCTTCATTGACAAGGT	Forward
	CGGGTGCCAACATTCATT	Reverse
SSTr2	AGCAACGCGGTCCTCACGTT	Forward
	GGAGGTCTCCATTGAGGAGG	Reverse
Nod2	GCTGTCTTGGGATGTGCT	Forward
	GGATGAAGGGAGTGAGTGTC	Reverse
Slpi	CTCAGGCAAGATGTATGATG	Forward
	TTTCCCACATATACCCTCAC	Reverse
Human syntenin	TTCTGCTCCTATCCCTCACG	Forward
	CCAGTTACAGGAGCCACCAT	Reverse
Hemicentin 1	ATGATTGCCCAGGAAGTG	Forward
	CTAGACATGGGTGGGGAA	Reverse
Hemicentin 2	ATGACGCCTGGGGCGCAG	Forward
	ATGAACTTTGGAGGCCTG	Reverse
E2F2	ACCGCCACCACCTACTACAC	Forward
	CCTCCAGGTCCAACTTCCTT	Reverse
Foxd1	TACTCGTACATCGCGCTCAT	Forward
	CTGCTGATGAACTCGCAGAT	Reverse
Hif1α	TGCTCATCAGTTGCCACTTC	Forward
	TGGGCCATTTCTGTGTGTAA	Reverse
Hif2α	TGAGTTGGCTCATGAGTTGC	Forward
	CTCACGGATCTCCTCATGGT	Reverse
Cxcl2	GAAGTCATAGCCACTCTCAAGG	Forward
	CCTCCTTTCCAGGTCAGTTAGC	Reverse

PRG2/MBP	CAAACTTGACAAGACCCAGGA	Forward
	GGACATCTGGCAGGAAAGAA	Reverse
Thbs1	TGGCCAGCGTTGCCA	Forward
	TCTGCAGCACCCCCTGAA	Reverse
Bmpr2	CTGCGGCTGCTTCGCAGAAT	Forward
	TGGTGTTGTGTCAGGAGGTGG	Reverse
MMP9	GTTTTTGATGCTATTGCTGAGATCCA	Forward
	CCCACATTTGACGTCCAGAGAAGAA	Reverse
Nptxr	GTGGAGAAGGAGCTGAATGC	Forward
	GGGCGTACATGTAGTTGTTGC	Reverse
PTHr	CAGCCGAAATCAGAGCTACC	Forward
	CTCCTGTTCTCTGCGTTTCC	Reverse
ND1	CGGGCCCCCTTCGAC	Forward
	GGCCGGCTGCGTATTCT	Reverse
ND2	CACGATCAACTGAAGCAGCAA	Forward
	ACGATGGCCAGGAGGATAATT	Reverse
Cyt C oxidase	CATCCCAGGCCGACTAAATC	Forward
	TTTCAGAGCATTGGCCATAGAA	Reverse
cyt b 245 heavy chain	TGCCAACTTCCTCAGCTACA	Forward
	GTGCACAGCAAAGTGATTGG	Reverse
NKX3-1	ATGCTTAGGGTAGCGGAGC	Forward
	TGCGGATTGCCTGAGTGTC	Reverse
Nfe2/NRF2	CTGAACTCCTGGACGGGACTA	Forward
	CGGTGGGTCTCCGTAAATGG	Reverse
Erbb4	CCCAGATCTCCACTGGCTCC	Forward
	TTCAGGGTTCTCCACAGCACC	Reverse
Erdr1	CTTTTAGCCGCAGCTATGGT	Forward
	ATTCACGCCCACAGAGAAAC	Reverse
TRP73	CTGGTCCAGGAGGTGAGACTGAG	Forward
	CTGGCCCTCTCAGCTTGTGCCACTTC	Reverse

CD300a	GCATTGTTGCTGCTTCTGTTG	Forward
	CACCAGCTTTGATCCATTTCTG	Reverse
CD300c	ACACCCTGGCTCCCTGTTC	Forward
	CTAGAGCTTCTCTGAGGTCTGTTCAC	Reverse
Ddx3y	AGAGGGTTTTCCAAGCGAGG	Forward
	TCGACCACTTCCACTTCTGC	Reverse
Rab44	AGAGACCACACACACTCTC	Forward
	CTCCTGTAAGTCTGTTCTTG	Reverse
Adam8	AGGATATTCAGCAGGTGTAGCAA	Forward
	TGCTAAAGGTATAGCAGGAGTCG	Reverse
Cadherin 9	GATCCGATTATCAGTACGTGGG	Forward
	TGTATGTCGCCTGTGTTCTC	Reverse
Disc1	TGGTCGAGGATGGCGATTACGA	Forward
	AGAGCAGGTTGCTGTGAAGGCA	Reverse
Ksr2	TGGATGTCCGAAAGGAAGTC	Forward
	CTTCTCCACGGTCTCACACA	Reverse
Urokinase receptor	TTCCACCGAATGGCTTCCAG	Forward
	AGGCAATGAGGCTGAGTTGAGC	Reverse
Kcna3/Kv1.3	TTGTGGCCATCATTCCTTA	Forward
	CCTGCTGCCCATTACCTTGT	Reverse
Xylt1	GTGGCATTCTCCACAGATGACC	Forward
	TTGCGGTTCCAGTTGGTGATGC	Reverse
Kcna3	TTGTGGCCATCATTCCTTA	Forward
	CCTGCTGCCCATTACCTTGT	Reverse
MAPK14	GATTCTGGATTTTGGGCTGGCTCG	Forward
	ATCTTCTCCAGTAGGTCGACAGCC	Reverse

Supplementary Table 4. Gene symbols for qRT-PCR validation

forkhead box D1(Foxd1)
hypoxia inducible factor 1, alpha subunit(Hif1α)
interleukin 1 beta(Il1β)
nucleotide-binding oligomerization domain containing 2(Nod2)
transformation related protein 73(Trp73)
cytochrome b-245, beta polypeptide(Cybb)
adenosine deaminase(Ada)
ArfGAP with coiled-coil, ankyrin repeat and PH domains 1(Acap1)
cadherin 9(Cdh9)
G protein-coupled receptor 132(Gpr132)
RAB44, member RAS oncogene family (Rab44)
DEAD (Asp-Glu-Ala-Asp) box polypeptide 3, Y-linked (Ddx3y)
thbsomatostatin receptor 2(Sstr2)
keratin 2(Krt2)
E2F transcription factor 2(E2f2)
chemokine (C-X-C motif) ligand 2(Cxcl2)
secretory leukocyte peptidase inhibitor(Slpi)
thrombospondin 1(thbs1)
Proteoglycan 2 (PRG2)
Parathyroid hormone 1 receptor (PTHr)
xylosyltransferase 1(Xylt1)
potassium voltage-gated channel, shaker-related subfamily, member 3(Kcna3)
neuronal pentraxin receptor(Nptxr)
matrix metallopeptidase 25(Mmp25)
kinase suppressor of ras 2(Ksr2)
disrupted in schizophrenia 1(Disc1)
a disintegrin and metallopeptidase domain 8(Adam8)

CD300 molecule (Cd300)

erythroid differentiation regulator 1(Erdr1)

erb-b2 receptor tyrosine kinase 4(Erbb4)

nuclear factor, erythroid derived 2(Nfe2/NRF2)

NK-3 transcription factor, locus 1 (Drosophila)(Nkx3-1)

cytochrome c oxidase subunit I(COX1)

NADH dehydrogenase subunit 2(ND2)

NADH dehydrogenase subunit 1(ND1)

plasminogen activator, urokinase receptor(Plaur)

neuronal pentraxin receptor(Nptxr)

matrix metallopeptidase 9(Mmp9)

bone morphogenetic protein receptor, type II (serine/threonine kinase)(Bmpr2)

Supplementary Table 5. Gene-Level Differential Expression Summary Table

ENTREZ ID	SYMBOL	GENENAME	logFC	AveExpr	t	P.Value	log10
**26900**	**Ddx3y**	**DEAD (Asp-Glu-Ala-Asp) box polypeptide 3, Y-linked**	**9.28778**	**1.92449**	**18.2606**	**2.30E-10**	**9.6392**
**20592**	**Kdm5d**	**lysine (K)-specific demethylase 5D**	**8.37495**	**0.47289**	**18.3596**	**2.15E-10**	**9.6675**
**26908**	**Eif2s3y**	**eukaryotic translation initiation factor 2, subunit 3, structural gene Y-linked**	**7.56009**	**0.98951**	**13.2715**	**1.02E-08**	**7.99237**
**22290**	**Uty**	**ubiquitously transcribed tetratricopeptide repeat gene, Y chromosome**	**6.4943**	**0.09791**	**11.6593**	**4.57E-08**	**7.3404**
**1E+08**	**Mir3960**	**microRNA 3960**	**3.0608**	**-1.86847**	**4.24352**	**0.00105**	**2.97837**
**16491**	**Kcna3**	**potassium voltage-gated channel, shaker-related subfamily, member 3**	**2.97393**	**-0.43315**	**3.06333**	**0.00056**	**3.24817**
**114640**	**Pth2**	**parathyroid hormone 2**	**2.92944**	**-0.51942**	**3.97632**	**0.0019**	**2.72195**
**233781**	**Xylt1**	**xylosyltransferase 1**	**2.84987**	**2.49826**	**3.13003**	**0.00834**	**2.07861**
**242705**	**E2f2**	**E2F transcription factor 2**	**2.72761**	**-0.34458**	**3.8923**	**0.00307**	**2.51247**
**27421**	**Abcc6**	**ATP-binding cassette, sub-family C (CFTR/MRP), member 6**	**2.70606**	**-1.3821**	**3.10565**	**0.00944**	**2.02487**

				2			
**546644**	**Ly6g**	**lymphocyte antigen 6 complex, locus G**	**2.55231**	**-2.34488**	**3.1121**	**0.00896**	**2.04767**
**319594**	**Hif1a**	**hypoxia-inducible factor 1, alpha subunit inhibitor**	**2.53759**	**4.86782**	**4.18753**	**0.00805**	**2.09419**
**628416**	**Gm6878**	**predicted gene 6878**	**2.50622**	**-2.30279**	**3.80096**	**0.00237**	**2.62555**
**442827**	**Rab44**	**RAB44, member RAS oncogene family**	**2.23999**	**-1.43793**	**2.4158**	**0.03325**	**1.47816**
**20568**	**Slpi**	**secretory leukocyte peptidase inhibitor**	**2.19406**	**-1.30544**	**4.00698**	**0.00162**	**2.79084**
**383326**	**Gm5237**	**predicted gene 5237**	**2.13247**	**-0.79592**	**2.95798**	**0.01202**	**1.92013**
**226654**	**Tstd1**	**thiosulfate sulfurtransferase (rhodanese)-like domain containing 1**	**2.06113**	**-0.13192**	**4.27205**	**0.001**	**3.0008**
**170942**	**Erdr1**	**erythroid differentiation regulator 1**	**-2.62243**	**-1.4716**	**-3.75986**	**0.00267**	**2.57297**
**213742**	**Xist**	**inactive X specific transcripts**	**-11.0481**	**-1.10652**	**-23.9543**	**8.52E-12**	**11.0693**

Online repository: **Sequence Read Archive (SRA)**

Accession number: **PRJNA1377687**
